# Overexpression of Glutamate Decarboxylase in Mesenchymal Stem Cells Enhances Their Immunosuppressive Properties and Increases GABA and Nitric Oxide Levels

**DOI:** 10.1371/journal.pone.0163735

**Published:** 2016-09-23

**Authors:** Mariana Urrutia, Sebastián Fernández, Marisol González, Rodrigo Vilches, Pablo Rojas, Manuel Vásquez, Mónica Kurte, Ana María Vega-Letter, Flavio Carrión, Fernando Figueroa, Patricio Rojas, Carlos Irarrázabal, Rodrigo A. Fuentealba

**Affiliations:** 1 Centro de Investigación Biomédica, Facultad de Medicina, Universidad de Los Andes, Santiago, Chile; 2 Departamento de Biología, Universidad de Santiago de Chile, Santiago, Chile; Michigan Technological University, UNITED STATES

## Abstract

The neurotransmitter GABA has been recently identified as a potent immunosuppressive agent that targets both innate and adaptive immune systems and prevents disease progression of several autoimmunity models. Mesenchymal stem cells (MSCs) are self-renewing progenitor cells that differentiate into various cell types under specific conditions, including neurons. In addition, MSC possess strong immunosuppressive capabilities. Upon cytokine priming, undifferentiated MSC suppress T-cell proliferation via cell-to-cell contact mechanisms and the secretion of soluble factors like nitric oxide, prostaglandin E2 and IDO. Although MSC and MSC-derived neuron-like cells express some GABAergic markers *in vitro*, the role for GABAergic signaling in MSC-mediated immunosuppression remains completely unexplored. Here, we demonstrate that pro-inflammatory cytokines selectively regulate GAD-67 expression in murine bone marrow-MSC. However, expression of GAD-65 is required for maximal GABA release by MSC. Gain of function experiments using GAD-67 and GAD-65 co-expression demonstrates that GAD increases immunosuppressive function in the absence of pro-inflammatory licensing. Moreover, GAD expression in MSC evokes an increase in both GABA and NO levels in the supernatants of co-cultured MSC with activated splenocytes. Notably, the increase in NO levels by GAD expression was not observed in cultures of isolated MSC expressing GAD, suggesting crosstalk between these two pathways in the setting of immunosuppression. These results indicate that GAD expression increases MSC-mediated immunosuppression via secretion of immunosuppressive agents. Our findings may help reconsider GABAergic activation in MSC for immunological disorders.

## Introduction

Mesenchymal stem cells (MSC) are multipotent, non-hematopoietic fibroblast-like cells with self-renewal capacity [1]. MSC cells participate in the regeneration of adult tissues by their ability to originate mesenchymal tissue such as bone, cartilage, muscle, tendon and adipose tissue [1–3]. These plastic-adherent cells do differentiate into osteoblastic, adipocytic, and chondroblastic lineages *in vitro* [4] and do express CD73, CD90 and CD105 markers, but not hematopoietic markers like CD14, CD34, CD45, and HLA-DR [5]. Low levels of cell-surface major histocompatibility complex molecules and lack of co-stimulatory receptors renders MSC cells evasive to the immune system [6,7]. A bulk of evidence now demonstrates they do indeed inhibit alloreactive T-cell responses [8–11]. Importantly, allogeneic human MSC do alleviate graft versus host disease [12,13]. Ongoing clinical trials for type 1 diabetes, acute myocardial infraction, multiple sclerosis, Crohn´s disease and systemic lupus erythematosus show promising effects in terms of immune modulation and safety [14,15], making MSC cells an attractive therapeutic tool for autoimmune diseases clinically relevant. Although preclinical data suggests that timing of MSC administration can severely affect outcome, switching MSC from an anti- to a pro-inflammatory regulator [16,17], MSC therapy for autoimmune diseases represents an emergent field with many possibilities from both translational and basic research perspectives [14,18]. Efforts towards identifying molecular pathways and druggable targets to improve MSC-mediated inhibition of the immune system represents a challenge and constitutes a hot research topic.

In order to become immunosuppressive, MSC require an activation step by the cytokines IFN-γ and either TNF-α, IL-1α, or IL-1β, stressing the need of an inflammatory milieu to become completely functional [19,20]. Evidence from animal studies and from *in vitro* experiments indicates that MSC-mediated immunosuppression takes place via both cell-to-cell contact mechanisms [19–22] and by the diffusion of MSC-secreted factors [23]. Among soluble mediators, nitric oxide plays an important role. In murine MSC, strong induction of iNOS gene expression do occur upon IFN-γ and TNF-α, IL-1α, or IL-1β co-treatment, and knockout experiments demonstrate requirement of MSC IFN-γ receptor and splenic IFN-γ genes for MSC-to-T-cell inhibition and nitric oxide secretion [20,24]. Genes for other secreted mediators are similarly regulated by pro-inflammatory stimulation in MSC, including PGE2 [19,25], HGF [9], TSG-6 [26,27] and HLA-G5 [28]. Importantly, species-specific mechanisms also operate, as depletion of the key metabolite tryptophan via induction of the catabolic enzyme IDO, but not iNOS induction, contributes to the mechanism for human MSC-mediated immunosuppression [21,29]. In all cases, evidence from knockout animals indicate that none of these soluble mediators works alone but a combination of effector molecules to modulate the immune system *in vivo* has been rather postulated [30,31]. The search for novel soluble factors for MSC-mediated immunosuppression is thus an area of intense research.

The neurotransmitter γ-aminobutiric acid (GABA) is a novel immune suppressor that targets both innate and adaptive immune systems [32]. GABA, synthesized from glutamate by glutamic acid decarboxylase (GAD), is the principal inhibitory neurotransmitter in the central nervous system (CNS)[33]. However, GABA synthesis and GABAergic signaling also occurs in the periphery. Detection of GABA and GAD enzymes has been reported in the pancreas [34,35], oviduct and testes [36,37], airway epithelia [38] and immune cells (reviewed in Prud’homme et al, 2015 [39]). Although the physiological role for peripheral GABA is not completely understood, it is now clear that either exogenously administered GABA, or elevation of endogenous GABA levels through pharmacological intervention promotes immunosuppression *in vivo*. GABAergic manipulation decrease the severity of symptoms in mouse models for autoimmune diseases type I diabetes [40,41], multiple sclerosis [42,43], rheumatoid arthritis [44] and type II diabetes induced by a high fat diet [45]. At the molecular level, T-cells do express functional ionotropic type A GABA receptors (GABA-A-R) both in humans [46–48] and rodents [48–50] and GABA or GABA-A-R agonists mediates the inhibition of T-cell proliferation and cytokine production [40,41,44,47,49,51]. This inhibitory effect presumably occurs via activation of outward chloride currents on lymphocytes cell membranes and consecutive impairment of calcium entry, which is required for T-cell proliferation [40,46,51]. Indeed, blocking of GABA-induced chloride currents with picrotoxin or bicucullin, two selective GABA-A-R antagonists [52,53], also antagonizes immunosuppression *in vitro* [46,48,49]. In addition to T-cells, functional GABA-A-R also exist in macrophages and dendritic cells, where GABA-A-R activation has been demonstrated inhibits LPS-induced IL-6, IL-12 and IL-1β cytokine production [41,42,54] as well as antigen presentation by antigen presenting cells [42,44]. Thus, mounting evidence demonstrates that GABA is a potent immunosuppressive agent with a wide range of immune cell targets.

A set of unrelated studies demonstrate that Bone-marrow derived MSC (BMMSC) do express functional GABA-A-Rs. BMMSC have long been clinically used for regenerative purposes of mesodermal tissue. However, under appropriate *in vitro* growing conditions BMMSC differentiate into cells derived from the ectoderm, including neurons [55,56]. Undifferentiated BMMSC cells do express GABA-A-R α1 and *in vitro* differentiation to a neuronal lineage induces the expression of all GABA-A-R α1, β3 and ε, as well as metabotropic GABA-B receptor subunit B [57]. In another study, GABA-A-Rs transcripts for α2 and β3 subunits have been detected in rat BMMSCs and protein levels of both subunits increased upon differentiation into a Schwann-like cell *in vitro* [58]. Of note, treatment of BMMSCs with muscimol, a specific GABA-A-R agonist, increases forskolin-induced proliferation rates of BMMSC-derived Schwann-like cells, indicating functional assembly of GABA-A-R on differentiated BMMSC to a glial lineage [58]. Finally, the group of Iwasaki and collaborators showed that BMMSCs from transgenic GFP mice injected into a stroke mouse model do express GABA-A-R α1 and MAP2 markers in the ischemic regions of the brain while only minor expression was detected in zones that are not damaged, like in the striatum [59]. The importance the authors give to these findings is that BMMSCs have the potential to become fully equipped neuronal and glial cells *in vitro*, perhaps compatible with *in vivo* neurotransmission and tissue regeneration. **However, evidence for GAD expression or GABA secretion by MSC has never been reported. Moreover, functional roles for GABAergic signaling in the context of their immunosuppression capacity remain elusive.** Here, we demonstrate that pro-inflammatory cytokines regulate GAD-67 expression in murine bone marrow-MSC. Gain of function experiments demonstrate that GAD overexpression increases immunosuppressive function in the absence of pro-inflammatory licensing. Moreover, GAD expression evokes an increase in both GABA and NO levels in the conditioned media of MSC co-cultured with CD3/CD28-activated splenocytes. Interestingly, the increase in NO levels by GAD expression was not observed in pure cultures of MSC expressing GAD, suggesting crosstalk between these two pathways in the setting of immunosuppression. Our findings may help reconsider GABAergic activation in MSC for immunological disorders.

## Materials and Methods

### Reagents and Antibodies

*From ThermoFisher Scientific (Waltham*, *MA*, *USA)*, MEM Alpha (#12571), RPMI 1640^®^ Glutamax (#72400), PBS 1x (#10010), Anti-GAD-67 Antibody (#PA521397), Cell Trace Violet kit (#C34557), Near IR DEAD/LIVE kit (#L10119), pcDNA3.3-TOPO-TA-clonining kit (#K830001), Trizol, DNAseI, PstI, RNAse A, 1kpb Plus DNA ladder, 6-well plates. *From Cell Signaling Technologies (Danvers*, *MA*, *USA)*, Anti-GAD65 Antibody (#5843S); From KPL (Gaithersburg, MD, USA), goat anti-rabbit-HRP (#474–1506), goat anti-mouse-HRP (#474–1806); *From Sigma-Aldrich (St Louis*, *MO*, *USA)*, AC15 Anti-β-Actin Antibody (#A1978), 2,3-diaminonaphthalene (#88461), alpha ketoglutaric acid (#K1875), NADP (#N5755), Resazurin (#R7017), 2-AEHS (#06720), DTT (#D0632), GABAse (#G7509), Diaphorase (#D5540), TEMED, TX-100, Tween20, EDTA, ammonium persulfate, ammonium chloride, sodium bicarbonate, sodium hydroxide. *From BD Biosciences (San Jose*, *CA*, *USA)*, Anti-CD34-FITC (#553733), Anti-CD45-PE (#553081), Anti-Sca-1-FITC (#557405), Anti-CD44-PE (#553134), Anti-CD29-FITC (#555005), Anti-CD4-PE-Cy5 (#553050), Anti-CD3ε (#553057), Anti-CD28 (#553294), Anti-iNOS (#610431), 20 mL, 10 mL and tuberculin syringes; *From Promega (Madison*, *WI*, *USA)*, Fugene 6, GoTaq Mastermix 2x, dNTPs, RNAsin, MMLV reverse transcriptase, Random Primers, Bradford Reagent; From SPL Life Sciences (Gyeonggi-do, Korea), T-25 and T-75 vent flasks, 48 well plates; *From Roche (Schweiz*, *Switzerland)*, Complete Inhibitor Cocktail EDTA free; From Millipore (Billerica, MA, USA), 0.45 μm PVDF membrane; *From GE Healthcare Lifeciences (Little Chalfont*, *UK)*, HyClone Serum (SH30396.03), films (#28906838); *From Stratagene (San Diego*, *CA*, *USA)*, PfuUltra II Fusion HS DNA polymerase (#600670–51), Polypropylene 96-Well tube plates, optical strip caps. *From R&D Systems (Minneapolis*, *MN*, *USA)*, rmIL-1β (401-ML), rmIFN-γ (485-ML). *From Biological Industries (Beit HaEmek*, *Israel)*, L-glutamine 100X, PenStrep 100X, Pyruvate 100X, Tripsin 10x; From Amresco (Solon, OH, USA), β-mercaptoethanol; *From Bio-Rad (Hercules*, *CA*, *USA)*, Tris, Glycine, Prestained dual color protein standard (#161–0374); *From Corning (Corning*, *NY*, *USA)*, solid black 96-well plates (Costar3915), Falcon^®^ Cell strainer 70 um; From MERCK (Kenilworth, NJ, USA) absolute ethanol, methanol, 2-propanol, HCl; *From QIAGEN (Hilden*, *Germany)*, QIAfilter Maxi kit (#12262); *From OMEGA BioTek (Norcross*, *GA*, *USA)*, Gel Extraction kit (#D2500-01); *From US BIO (Washington*, *DC*, *USA)*, Ampicillin, SDS; *From MO BIO (Vancouver*, *Canada)*, LB broth (#12106–1), LB agar (#12107–05).

### Plasmids

Dr. Allan Tobin and Dr. Niranjala Tillakaratne at UCLA kindly provided plasmids pGEX-3X+HGAD67 (1D) and pGEX-3X+HGAD65 (2E) [60]. Human GAD-67 and GAD-65 coding sequences were PCR amplified using PfuUltra II Fusion HS DNA polymerase (Agilent Technologies). Primers used were: 5’-GAT CGG ATC CAC CGG TAC CGA GCT GAT GGC GTC TTC GAC CCC ATC T-3’ (GAD-67-S), 5’-GAT CGA ATT CGA TTA CAG ATC CTG GCC CAG TCT T-3’ (GAD-67-AS), and 5’-GAT CGG ATC CAC CGG TCC AAA GCC GAT GGC ATC TCC GGG CTC TGG-3’ (GAD-65-S), 5’-GAT CGA ATT CGT TAT TAT AAA TCT TGT CCA AGG CGT TC-3’(GAD-65-AS). After PCR amplification, adenine residues were added by incubation with TaqPolymerase and purified fragments were subcloned into pcDNA3.3-TOPO TA (Invitrogen) as recommended by the manufacturer. After selecting proper orientation by restriction enzyme analysis (*Pst*I, Fermentas), selected colonies were double-strand sequenced to verify identity. Immunodetection of 67 and 65 kDa proteins, and detection of secreted GABA levels in transfeceted HEK293T confirmed proper cloning and expression.

### MSC cell culture

MSC were grown in MEM Alpha media supplemented with 10% FBS, 1% Pen/Strep and 2 mM L-glutamine. MSC cells were regularly plated at 4,800, 12,000 or 24,000 cel/cm^2^ in T-25 vent flasks for 3, 2 or 1 day of growth, respectively. Our seeding protocol guaranteed reaching <80% confluency at the time of analysis or procedure. For tripsinization, cells were washed twice with PBS 1x and tripsinized by incubation for 5 min using 1 mL of tripsin 1x. Cells were then dislodged mechanically by gently shaking the flasks followed by passing the cells 10 times through a P1000 tip.

### MSC licensing

For cytokine priming, cells were seeded at 4,800 cel/cm^2^ in 4 mL complete media. 24 h later, 1 mL of growing media was removed and the growing media was supplemented with 10 ng/mL IL-1β (2 ng/μL stock) and/or 25 ng/mL IFN-γ (25 μg /μL stock). Cultures were immediately returned to the incubator and cells were additionally grown for 48 h. Staggered experiments were performed when comparing 24 h priming, in which case cells were identically treated but addition of cytokines were performed 48 h after seeding. At end point, conditioned media was collected and cells either tripsinized for immunosuppression assays or lysed for Western blot experiments.

### MSC transfection

In GAD-67/GAD-65 co-transfection experiments for GABA secretion optimization, cells were seeded at 10,000 cells/cm^2^ in 6 well plates using 2 mL complete media. 24 h later, cells were transfected with a total amount of 0.5 μg DNA using Fugene 6 reagent in a 1:3 ratio as recommended by the manufacturer, with modifications. Briefly, 0.5 μg DNA were placed at the bottom of a 1.5 mL Eppendorf tube (RunnLab) and DMEM was added to complete 50 μL. Then 1.5 μL of Fugene6 was swirlingly added to the diluted DNA mixture avoiding touching the plastic, and contents mixed thoroughly. Mixtures were incubated for 15 minutes at room temperature for complex formation and then applied on cells dropwise.

For immunosuppression assays, cells were seeded at 12,000 cel/cm^2^ in T-25 vent flasks. 24 h later cells were transfected using Fugene 6 reagent as described before but complexes were prepared in a final volume of 130 μL and 5.2 μg total DNA were used. Where indicated, 10.4 μg total DNA was also used (2X).

### Animals

C57BL/6 mice, 8–14 weeks old, were purchased from the Central Animal Facility, Faculty of Medicine, University of Chile. Experimental procedures and protocols were performed according to the US National Institutes of Health Guide for the Care and Use of Laboratory Animals (NIH publication number 85–23, revised in 1996) and were approved by the Institutional Animal Care and Use Committee of the Universidad de los Andes and the CONICYT-Fondecyt Bioethics Advisory Committee in Chile. Animals were applied an overdose of ketamine/xylazine and verified completely non-responsive to noxious stimuli before euthanasia by cervical dislocation.

### Isolation of splenocytes and TCD4+ cells, and labeling with Cell Trace Violet

12- to 15-wk-old C57BL/6 male mice were sacrificed and splenocytes were isolated by gently pressing spleens through a 70-μM mesh cell strainer. Splenocytes were then ACK-lysed for exactly 2 min on ice using pre-chilled ACK 1x pH = 7.15, and extensively washed in PBS1x (50 mL). ACK-treated splenocytes were either resuspended in room temperature PBS1x for Cell Trace Violet^®^ labeling (Invitrogen), or in cold PBS containing 2% FBS and 2 mM EDTA (Isolation Buffer) for TCD4+ lymphocytes isolation using the mouse untouched TCD4+ kit (Invitrogen).

To isolate TCD4+ cells, ACK-treated splenocytes were brought to 100,000,000 cells/mL in cold Isolation Buffer and 0.5 mL of this suspension was placed in a sterile 15 mL conical tube. The suspension was supplemented sequentially with 100 μL room temperature FBS and 100 μL cold antibody mix and cells were incubated for 20 minutes on ice. 10 mL Isolation Buffer was then added and cells were pelleted by centrifugation for 8 min at 350 *xg*, 4°C to remove the excess of antibodies. Cells were then resuspended in 4 mL cold Isolation Buffer and 1 mL pre-equilibrated beads were added. Bead-to-cell complexes were allowed to form by gently mixing the contents in a rotamix for 15 minutes at room temperature. Finally, 5 mL of room temperature Isolation Buffer were added and T cells were recovered in the supernatant by negative selection by applying the mixture to a magnet (Invitrogen). Unbound TCD4+ cells were then collected by centrifugation (10 min at 350 *xg*) and resuspended in 0.5 mL room temperature PBS for Cell Trace Violet labeling.

For splenocyte labeling, 15x10^6^ splenocytes were brought to 3 mL in PBS. 3.75 μL Cell Trace Violet^®^ 5 mM were then pre-diluted in 1 mL PBS1x and ~1 mL diluted CTV was quickly and swirlingly added to the splenocyte suspension to homogeneously mix the contents. Cells were incubated 10 minutes in a cell incubator, mixed by inversion and returned to the incubator for additional 10 minutes. After completing 20 minutes labeling, Quenching media (any RPMI media supplemented with 10% FBS) was added to complete 50 mL and the suspension was incubated additional 5 minutes in the incubator in order for serum proteins compete for labeling of cellular proteins. Cells were finally pelleted and resuspended in 500 μL Complete media (RPMI 1640/Glutamax^®^, supplemented with 1% Pen/Strep, 2 mM Glutamine, 1 mM Sodium pyruvate and 55 μM βME). For TCD4+ cell labeling, an identical protocol was followed but 5x10^6^ TCD4+ cells were brought to 1 mL PBS and 1.25 μL Cell Trace Violet^®^ 5 mM was used instead in the first step. In all cases, total amount of cells, CTV reagent and final volume were scaled up or down accordingly, but pre-diluted CTV was always applied as 1 mL addition. Finally, cells were counted and seeded in Anti-CD3ε pre-coated 48 well plates as described below.

### Immunosuppression assay setup and conditioned media collection

One day before co-culture setup, 48 well plates were coated with Anti-CD3 by adding 150 μL Anti-CD3ε pre-diluted 1:1,000 in PBS1x. After 30 min incubation in the cell incubator, treated plates were carefully sealed with parafilm, wrapped in plastic foil, and transferred to the fridge. Next day, CTV-labeled cells were counted and diluted properly in Complete media. Right before adding the cells, 48-well plates were washed twice with Complete media (5 min incubator, 500 μL). Cells were seeded in a final volume of 300 μL and 4 μL Anti CD28 antibody diluted 1:10 in Complete media was added immediately. ~3 hours after seeding, MSC were tripsinized and added at indicated doses in a volume of 100 μL, to make a final volume of 400 μL. For assays that required measurements of GABA and nitric oxide, seeding was identically performed but 400 μL splenocytes and 5 μL diluted Anti CD28 were used instead. Introducing this minor change in volume did not compromise immunosuppression properties. Co-cultures were developed for 60 hours, and then conditioned media was collected for GABA and nitric oxide determinations, and cells detached for T-cell proliferation assessment by FACS.

For GABA and nitric oxide determinations, conditioned media from co-cultures were collected as follow. 280 μL conditioned media were transferred to an 1.5 mL Eppendorf tube (Runnlab) and clarified by centrifugation for 10 minutes at 400 *xg*, 4°C. 70 μL clarified conditioned media were transferred to a new 1.5 mL Eppendorf tube for denaturation as indicated in GABA assay section, and 180 μL were transferred to another 1.5 mL Eppendorf tube, combined with 180 μL RPMI1640 Glutamax^®^ media and probed for nitric oxide detection.

### Proliferation assessment

60 h after immunosuppression setup, cells were gently resuspended in the same growing media and transferred to a 5 mL flow cytometry tube. Cells were washed once with PBS and probed with 100 μL LIVE/DEAD^®^ near infrared dead cell stain (1:500) for 30 minutes at 4°C in the dark. After a PBS wash, cells were probed with 50 μL Anti-CD4-PE Cy5 antibody (1:20) for 20 minutes at 4°C in the dark. Cells were finally washed with PBS, centrifuged, resuspended in 300 μL and cells analyzed by FACS using a FACSCanto II^®^ cytometer coupled with a FACSDiva software. Gating strategy included (1) FSC versus SSC, (2) LIVE/DEAD^®^ near infrared versus SSC to select viable cells, (3) PE-Cy5 versus SSC to select viable TCD4+ cells, and (4) cell histograms for Pacific Blue channel (Cell Trace Violet) on viable TCD4+ cells. Cell proliferation was computed by calculating Proliferation Indexes using established algorithms [61]. Briefly, for each sample, markers for each one of the CTV peaks were set, and absolute number of cells at each peak was determined. Then, absolute number of precursors at each peak were calculated by dividing this number by 2^*n*-1^ being *n* the peak number. The sum of all progenitor cells calculated from every peak represents the progenitor population of cells. Mitotic cells were then calculated at every peak by subtracting the number of progenitor cells from total events in the corresponding peak, and proliferation index was calculated by dividing the sum of all mitotic events by the number of absolute precursors. To decrease scattering of data between experiments, Proliferation indexes of stimulated lymphocytes in the absence of MSC were assigned an arbitrary value of 100%. For proliferation assays where conditioned media was removed for detection of analytes, 0.5 mL PBS were added prior to cell resuspension to facilitate dislodging of cells.

### Real Time PCR

Total RNA was extracted from cultured MSC using Trizol reagent (Invitrogen) and treated with DNAse I (Fermentas). Two μg of DNAse I-treated RNA was reverse-transcribed using ImProm RT and random hexamers (Promega) in 30-μL total volume reaction according to the manufacturer’s recommendations. PCR was performed using 2.5 μL of diluted cDNA (1: 100–1: 500) and 10 μL of primer-containing GoTaq Master Mix (Promega, 150–600 nM each primer) and analyzed using Mx3000P qPCR system (Agilent Technologies). Primers used were as follows: 5’-AAG GAC CAA TAG CCT GGA AGA -3’ (GAD-67, sense), 5’-GTT GGA GAA GTC GGT CTC TGT -3’ (GAD-67, antisense), 5’-TGC TTC AGT ACG TGG TGA AAA G -3’ (GAD-65, sense), 5’-TCC TCC AGA TTT TGC GGT TGG -3’(GAD-65, antisense), 5’-AAG AGA GCA GAG GTA ACT ACC T -3’ (ABAT, sense), 5’-GCT CGC GTT CTG AGG CTG TTG -3’ (ABAT, antisense), 5’-CGG ACA GGA TTG ACA GAT TG -3’ (18S, sense), and 5’-CAA ATC GCT CCA CCA ACT AA -3’ (18S, antisense). Efficiencies were 93.1% (GAD-67; 600 nM), 95.2% (GAD-65; 300 nM), 95.6% (ABAT; 300 nM) and 96.7% (18S; 150 nM) and cycling conditions were: Segment 1 (denaturation), 2 min 95°C; Segment 2 (x40 cycles), 15 sec 95°C, 45 sec 61°C, 45 sec 72°C; Segment 3 (dissociation curve), 15 sec 95°C, 1sec 25°C, 15 sec 70°C, 1 sec 90°C. As a positive control, whole brain cDNA was used as a template to corroborate amplicon identity by dissociation curve analyses and gel electrophoresis. Expression level of transcripts was normalized to 18S mRNA levels (normalizer) and to control healthy mice (control) according to the standard 2^-ΔΔCt^ method.

### Western blotting

Cells were lysed as described before [62]. Briefly, cells were washed twice in PBS and cell lysates prepared in 120 μL PBS containing 1% TX-100, supplemented with 1 mM PMSF and a protease inhibitor cocktail. After 30 min on ice, cells were vortexed for 12 seconds and cell debris discarded by centrifugation (10,000 *xg* for 10 min at 4°C). 30 μg of protein were resolved by 8% SDS-PAGE and transferred to PVDF membranes for 2 hours. After the membranes were blocked, proteins were detected using commercial antibodies GAD-67 (1:2,000), GAD-65 (1:1,000), iNOS (1:1,000), and β-actin (1:40,000) overnight at 4°C. The immunoblotting was followed by detection with a horseradish peroxidase-conjugated secondary antibody and enhanced chemiluminiscence substrate. Bands on films were scanned and quantitated using Image J software when needed. For detection of iNOS in the co-culture setting, two wells per condition were used. Lysis buffer was used to scrape the bottom of corresponding wells for full recovery of MSC-derived proteins, before combining to mechanically detached, pelleted cells.

### GABA levels determination

A recently developed protocol for GABA determination couples reduction of resazurin to enzymatic reactions initiated by GABA transamination [63]. Briefly, 250 μM Resazurin (4°C), 100 mM sodium α-ketoglutarate (-80°C), 5 mM NADP (-80°C) and 1 mM DTT (-80°C) stock solutions were prepared as recommended by Dr. Ippolito (personal communication). A mastermix designed to contain 6.25 μM Resazurin, 5 mM α-ketoglutarate, 100 μM NADP, 3.125 μM DTT and 0.0625 U/mL both GABAse and Diaphorase enzymatic activities in a Tris buffered solution were divided in two, and accordingly supplemented with 50 mM 2-AEHS or water. Samples and standard curve samples were plated in a volume of 10 μL on 96-well solid black plates and 90 μL of each mastermix were separately added to start the reaction. After 30 min incubation at room temperature, fluorescence was determined in an Infinite M1000 plate reader (Tecan Group Ltd, Männedorf, Switzerland) using Top measurements. Parameters were: Multiple Reads per Well (Circle (filled)), 3 x 3; Multiple Reads per Well (Border) 500 μm; Excitation Wavelength, 560 nm; Excitation Bandwidth, 10 nm; Emission Wavelength, 590 nm; Emission Bandwidth, 20 nm; Gain 60 (Manual); Number of Flashes, 50; Flash Frequency, 400 Hz. The fluorescence intensity values obtained from mastermixes +/- 2-AEHS were subtracted to calculate GABA-specific signal as described [63] and values interpolated in standard curves prepared in matching media.

To avoid signal contamination by enzymatic activity present in the samples, cleared conditioned media was denatured prior to measurement, as recommended [63]. MSC conditioned media from GAD-transfected cells were denatured as follow: 300 μL cleared conditioned media were dispensed in a 1.5 mL microtube (Runnlab) and 50 μL HCl 350 mM were added. After a little vortex step, samples were incubated in a thermoblock at 60°C for 30 minutes, spun down and neutralized by adding 50 μL Tris Base 400 mM. Samples were briefly mixed by vortex and centrifuged for 5 minutes at 15,000 *xg*, 4°C. Denatured samples were immediately measured or stored at -80°C until used. Co-culture conditioned media were similarly denatured but 70 μL of co-culture supernatant, 11.67 μL HCl and 11.67 μL Trisma Base were used.

### Nitric Oxide determination

We implemented the 2,3-diaminonaphthalene (DAN) assay to detect nitrites according to the method by Misko et al. (1993) and Kleinhenz et al. (2003) [64,65] with only minor modifications. In brief, DAN was dissolved in 0.62 N HCl at a concentration of 0.025 mg/ml. Supernatants from cultured cells were recovered and clarified by centrifugation at 400 *xg* for 10 minutes at 4°C. In order to avoid β-mercaptoethanol interference, co-culture supernatants were diluted 1:2 with RPMI1640 alone before measurement. Standard curves were prepared in matching media, i.e. DMEM + 10%FBS + L-Gln and Pen/Strep for pure MSC cultures or Complete Media:RPMI1640 (1:1) for co-culture experiments. Stock solution of nitrite was 20 mM in Mili-Q water, and it was diluted 1:100 in matching media to obtain a 200 μM working dilution, and then 1:10 as the first point of a two-fold serial dilution curve covering 10 data points. Dilution buffer and blanks were either DMEM- or RPMI-based media, depending on the corresponding experimental setting. Aliquots of sample supernatants and standard curves (100 μL) were placed into solid black Costar 3915 96-well plates (in triplicate), combined with DAN (20 μL) and incubated for 15 min at 37°C in the dark. After 15 min, 20 μL of 0.7 N NaOH was added to each well. Samples were analyzed for fluorescence using an Infinite M1000 plate reader (Tecan Group Ltd, Männedorf, Switzerland) using Fluorescence Top Reading. A full detailed protocol with detection settings and expected results is shown in S1 Protocol.

## Results

### Immunosuppressive properties of commercial MSC and priming with IL-1β and IFN-γ

**Fig 1:**
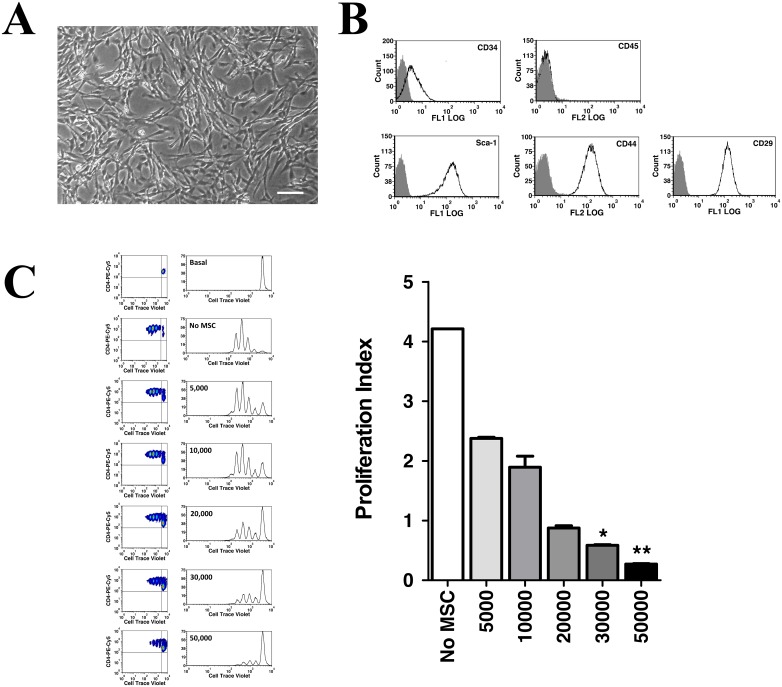
Characterization of commercial murine mesenchymal stem cells. C57BL/6 MSCs (Invitrogen, # S1502-100) were cultured as recommended by the manufacturer and utilized below passage 18. (A) Morphology of MSCs in culture observed with ×40 magnification on an inverted phase-contrast microscope. MSC cells present with a characteristic spindle-shape morphology. Scale bar, 100 μm. (B) Proper stem cell markers expression in cultured MSC. On passage 18, MSC were immunotypified for CD34, CD45, Sca-1, CD44 and CD29. MSC showed negative for hematopoietic markers CD34 and CD45 and positive for Sca-1, CD44 and CD29. Histograms for isotype controls are shown in grey. (C) Invitrogen MSCs are immunosuppressive. CD4 T-cells were isolated from healthy C57BL/6 mice using CD4+ untouched kit (Invitrogen) and labeled with Cell Trace Violet^®^ (CTV; Invitrogen) for proliferation analysis. 300,000 CTV-labeled CD4+ T-cells were stimulated with anti-CD3/anti-CD28 in the presence of indicated amounts of MSC and T-cell proliferation was assessed by flow cytometry 60 h later, as described in *Methods*. *Left Panel*, representative histograms from T-cell cultures showing dilution patterns of CTV signal in CD4^+^-gated cells. To localize the pool of non-proliferating cells with undiluted CTV, control cultures were kept not stimulated (basal). Proliferation indexes were calculated from cell events at each dilution peak using described algorithms. MSC cause a dose-dependent decrease in T-cell proliferation. *, p<0.05 (Kruskal-Wallis test with Dunn’s correction, N = 3).

**Fig 2:**
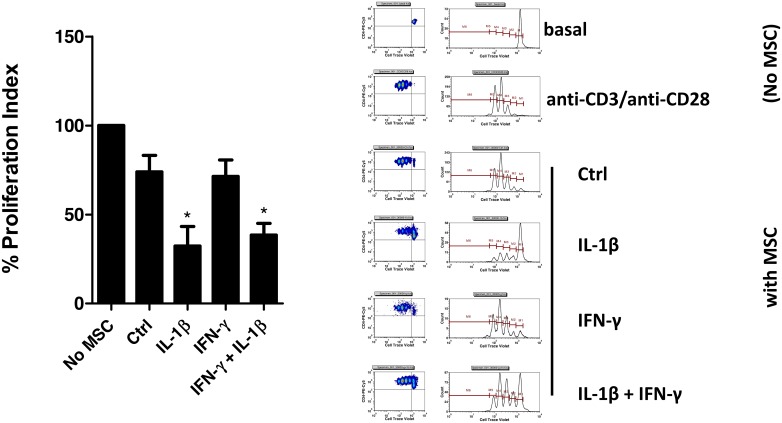
IL-1β pre-treatment increases immunosuppressive properties of MSCs. MSC were seeded at 4,800 cells/cm^2^ and grew for 24 h. MSC cultures were then supplemented with 20 ng/mL IL-1β, 25 ng/mL IFN-γ, or the combination of both cytokines as described in *Methods* and MSC cells were grew for additional 48 h. The ending day for the cytokine treatment, splenic TCD4+ lymphocytes were isolated from healthy C57BL/6 mice, labeled with CTV for proliferation assessment and stimulated with anti-CD3/anti-CD28 for 4 h. MSC were then added to CTV-labeled TCD4+ cells (ratio 1:10 MSC:TCD4^+^) and co-cultures were continued for additional 60 h as described in *Methods*. Proliferation controls included the determination of background and maximal proliferation for the T-cell preparation (basal, No MSC and anti-CD3/anti-CD28, respectively), and basal MSC-mediated immunosuppression was determined using untreated MSC (MSC Ctrl). Priming with IL-1β increases immunosuppressive properties of MSC. *, p<0.05 (Kruskal-Wallis test with Dunn’s correction, N = 3). *Right Panel*, representative histograms showing gating strategy and CTV dilution peaks for each treatment.

GIBCO^®^ Mouse (C57BL/6) MSCs is a commercial preparation of bone marrow-derived MSC obtained from young, healthy female mice that is commercialized at passage 8. Although stem cell properties of this MSC preparation have been demonstrated, immunosuppressive properties have never been confirmed. We first confirmed suitability of our growing conditions to properly expand MSCs by verifying adult stem (CD29, CD44 and Sca-1) and negative hematopoietic (CD34, CD45) cell markers by flow cytometry. In our cell culture conditions, Invitrogen MSCs presented readily adherent with a distinctive spindle-like, fibroblastic morphology (Fig 1A) and were positive for CD44, CD29 and Sca-1 markers, while negative for CD45 and CD34 (Fig 1B). Slight positivity for CD34 was readily detected, but this has been previously described for murine-derived MSC, especially in the C57BL/6 genetic background [66]. Therefore, our growing conditions demonstrate appropriate for growing and expanding MSC in cell culture. We next confirmed immunosuppressive properties of MSCs by evaluating the inhibition of T-cell proliferation in co-culture experiments using Cell Trace Violet-labeled TCD4+ cells and flow cytometry analysis. We observed a dose-dependent inhibitory effect on T-cell proliferation (Fig 1C) and strong immunosuppressive capacity of MSCs above ratios 1:10, as previously described for both human and murine MSCs [8,9,23,24,67]. Therefore, commercially available MSCs are suitable for immunological studies, as expected. We next characterized the effect of pro-inflammatory cytokines in activating MSC-mediated immunosuppression, a well-described phenomenon also known as *priming* or *licensing* of MSC [20,67–71]. We pre-treated MSCs with IL-1β and IFN-γ, either alone or in combination, for 24 h (S1 Fig) or 48 h (Fig 2) and the immunomodulatory properties of MSC were assessed by immunosuppression assays using isolated TCD4+ cells as before. Consistent with previous reports, MSCs are not strongly immunosuppressive in basal conditions, and treatment with IFN-γ alone was not sufficient to increase MSC immunosuppressive capabilities (Fig 2A) [20]. On the contrary, co-treatment of IFN-γ and IL-1β enhanced immunosuppressive properties of MSC. Surprisingly, a significant increase of immunosuppressive properties was also obtained by treatment with the cytokine IL-1β alone (Fig 2A). T-cell proliferation was decreased from 73.98±9.26% to 32.29±10.94% using IL-1β-treated MSC, compared to untreated MSCs. It is unlikely that the observed drop in T-cell proliferation would be a simply consequence of increasing TCD4+ cell death, as proliferation data was analyzed on gated, viable T-CD4+ cells and well-defined peaks for proliferation in CTV histograms were detected (Fig 2B). These results may imply that enough IFN-γ signaling activation was achieved in our co-culture conditions. Indeed, co-licensing of MSCs with IL-1β and IFN-γ did not cause a further increase in the immunosuppressive properties of MSC, compared to IL-1β only treatment. To confirm biological activity of recombinant IFN-γ, we took advantage of the strong effect combined cytokines have on inducible nitric oxide (iNOS) gene expression regulation in murine MSCs in the absence of T-cell co-culture [20]. We treated MSCs with IL-1β and IFN-γ for 24 or 48 h in the absence of T-cells, and we determined both cellular iNOS protein levels by Western blot and secreted nitric oxide levels in the conditioned media using a fluorescent-based method (DAN assay; [64]). Detection of increased levels of iNOS protein (S2A Fig) and secreted nitric oxide (S2B Fig) in MSC treated with IL-1β and IFN-γ verifies suitability of the cytokines used in this study, and corroborates cell responsiveness of commercial MSCs to IFN-γ. Collectively, these experiments demonstrate that we were able to properly expand commercially available murine MSCs in cell culture, to confirm their *in vitro* immunosuppressive properties and to describe a previously unreported effect for IL-1β alone to prime murine MSCs.

### Pro-inflammatory cytokines IL-1β and IFN-γ regulate GAD-67 gene expression

**Fig 3:**
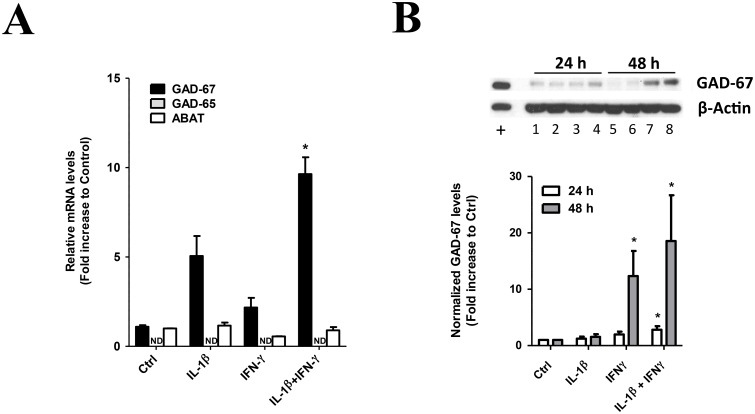
GAD-67 gene expression regulation by pro-inflammatory cytokines IL-1β and IFN-γ in MSCs. MSC were seeded at 4,800 cells/cm^2^ and grew for 24 h. MSC cultures were then supplemented with 20 ng/mL IL-1β, 25 ng/mL IFN-γ, or the combination of both cytokines, and MSC cells were grew for indicated times. Control cultures were identically treated but cytokines were not added. (A) GAD-67, GAD-65 and ABAT mRNA levels were determined 48 h after cytokine treatment by RT-qPCR. IL-1β selectively increases GAD-67 mRNA levels in MSCs and IFN-γ potentiates IL-1β effects. *, p<0.05 (Kruskal-Wallis test with Dunn’s correction, N = 3). (B) Sister cultures of experiments as in A were set and protein lysates prepared at 24 or 48 h after cytokine treatment. Proteins (30 μg) were subjected to 8% SDS-PAGE and GAD-67 protein levels were determined by Western blot using β-actin as a loading control. 15 μg of total brain lysate was used as a positive control. *Lower panel*, densitometric analysis of Western blot results. *, p<0.05 (Kruskal-Wallis test with Dunn’s correction, N = 11).

Whether MSCs use GABA as an immunosuppressive agent is presently unknown. In order to test this hypothesis, we reasoned that those conditions that increase immunosuppressive properties of MSC should also modulate biosynthetic and degradative machineries for GABA. The expression of GABA biosynthetic enzymes GAD-67 and GAD-65 has never been addressed in MSCs. First, we characterized GAD-67, GAD-65 and ABAT mRNA levels by RT-qPCR in both basal and cytokine-treated MSCs (Fig 3A). Basal expression was readily detected for both GAD-67 (Ct≈30) and ABAT (Ct≈23), and pro-inflammatory cytokines caused a selective increase in GAD-67 mRNA (Fig 3A). Interestingly, the increase in GAD-67 mRNA levels occurred not only using combined IL-1β and IFN-γ priming but also using IL-1β treatment alone, the two conditions that also increased immunosuppressive properties of MSC. To demonstrate whether increased mRNA levels translate to increased GAD-67 protein levels we analyzed GAD-67 expression by Western blot (Fig 3B). Surprisingly, IFN-γ but not IL-1β treatment were important to increase GAD-67 protein levels, with maximal increase of GAD-67 protein levels obtained using a combination of both IL-1β and IFN-γ. These results suggest a complex mechanism for GAD-67 gene expression regulation by pro-inflammatory cytokines and demonstrate that IL-1β and IFN-γ causes a significant increase in both GAD-67 mRNA and protein levels in cultured MSCs. In our experimental conditions GAD-65 was never detected, neither by RT-qPCR (Fig 3A) nor by Western blot (S3 Fig). Our results indicate that priming conditions that increase immunosuppressive properties of MSC selectively increases mRNA transcripts for GAD-67, suggesting that GAD expression and GABA levels might be important for MSC-induced immunosuppression.

### GAD-67/GAD-65 overexpression increases GABA secretion and immunosuppression capabilities of MSC

Our observation that priming conditions that do increase the immunosuppressive properties of MSC also increased GAD-67 mRNA levels, prompted us to determine whether GAD-67 expression was involved in the immunosuppression capacity of MSC. To address this, we choose a gain of function strategy instead of a knockdown approach. The main reason for this choice is that, upon triggering knockdown machineries (siRNA, shRNA), mammalian cells do activate expression of type I interferons presumably via TLR3 signaling activation, with a concomitant increase in IFN-β expression [72,73,74,75]. As MSCs do express TLRs involved in double stranded RNA sensing, like TLR3, TLR7 and TLR9 [76,77,78,79], and given that TLR3 activation increase immunosuppressive capabilities of murine and human MSC [80,81,82], we preferred to skip loss of function analyses to avoid possible masking artifacts on the immunosuppressive capabilities when using GAD-67 RNAi. We therefore utilized overexpression of GAD as an alternative approach. We rationalized that, in the absence of any pro-inflammatory licensing, the solely expression of GAD should increase MSC-mediated inhibitory effects on T-cell proliferation in co-culture. Because mechanisms of GABA secretion by MSCs have never been addressed, we first characterized GABA secretion in GAD-transfected MSCs.

**Fig 4:**
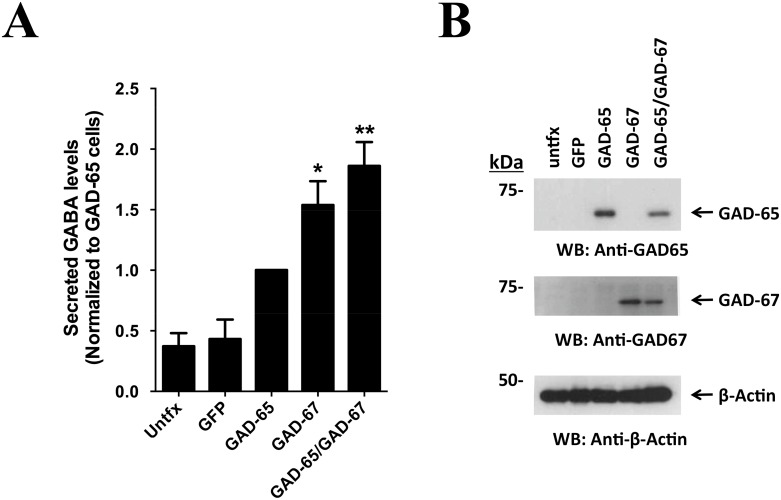
Increased GABA secretion with GAD-65/GAD-67 co-expression. MSCs were seeded at 10,000 cells/cm^2^ in 6 well plates and grew overnight. Cells were then transfected with Fugene6 as described in *Methods* using 1 μg total plasmid, and cells were recovered for 24 h. Co-expression with GAD-65 and GAD-67 used equivalent amounts of each plasmid. (A) Conditioned media was pre-cleared by centrifugation and secreted GABA levels were enzymatically determined using a fluorescence-coupled assay. Basal levels were 2.48 ± 0.83 μM and results were expressed as fold change to GAD-65 transfected (GAD-65) cells. Maximal secretion of GABA was detected using GADs co-expression. *, p<0.05 (Kruskal-Wallis test with Dunn’s correction, N = 5). (B) 15 μg (GAD-65, β-actin) or 30 μg (GAD-67) proteins were separated by 8% SDS-PAGE, proteins transferred to PVDF membranes and GAD-65, GAD-67 or β-actin immunodetected by Western blotting. Note that GAD levels in co-transfected cells were approximately half the levels of GADs expressed individually.

Evidence from knockout animals indicate that GAD-67 plays a key role in the maintenance of brain GABA levels [83,84,85,86], and biochemical and crystallographic evidence suggests that GAD-67, unlike GAD-65, is a constitutively active enzyme [87,88]. Although neuronal GAD-67 possesses both GAD-65 dependent and independent mechanisms for its anchoring to the plasma membrane for GABA secretion [89, 90, 91], evidence for the expression of GAD isoforms and their consequences on secreted GABA levels in non neuronal cells is scarce. Thus, we transfected MSCs with GAD-65, GAD-67 or a combination of GAD-65/GAD-67 (GADs) and determined GABA levels in the conditioned media 24 h later using a fluorescence-based assay [63]. Untransfected and GFP-transfected MSC were used as negative controls. As indicated in Fig 4A, untransfected MSCs presented secreted GABA levels of 2.48±0.83 μM, and GFP expression raised these backgrounds levels minimally (2.81±0.84 μM). A 3.8 fold increase in secreted GABA levels was detected in GAD-65 expressing cells (9.27±1.41 μM), while GAD-67 expression increased secreted GABA levels by 5.2 times as compared to control (14.63±1.61 μM). Finally, expression of combined GADs cause a further increase in GABA secretion compared to cells transfected with single GAD isoform, reaching 7.5-fold increase over basal MSCs (16.61±3.53 μM). To confirm proper expression of both isoforms, protein levels were confirmed by Western blot (Fig 4B). Approximately half levels of each enzyme were detected in doubly transfected cells. Thus, co-expression of GAD-67 and GAD-65 isoforms in MSCs boosted steady state levels of extracellular GABA levels compared to each isoforms expressed singly.

**Fig 5:**
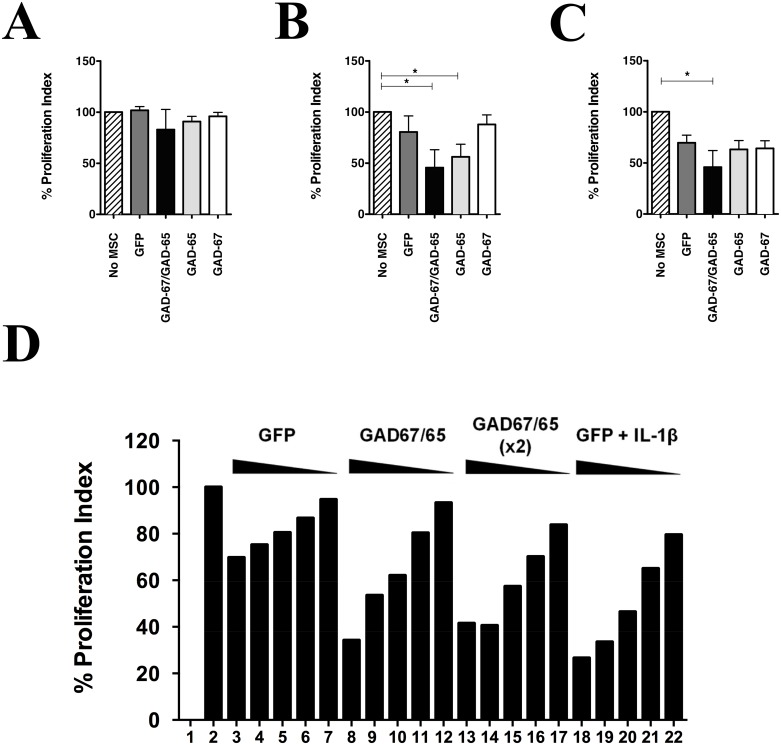
GAD-65/GAD-67 co-expression increases MSC immunosuppression. MSCs were seeded and transfected as indicated in Fig 2. Where indicated, IL-1β treatment was initiated right after addition of DNA complexes. 24 h after treatment, MSC:TCD4+ co-cultures were assembled at MSC:TCD4+ ratios of (A) 1:30; (B) 1:15; or (C) 1:10, and cell proliferation was assessed by FACS 60 h later as before. GADs co-expression increases immunosuppressive properties of MSC. *, p<0.05 (Kruskal-Wallis test with Dunn’s correction, N = 3). (D) Dose-dependent inhibition of T-cell proliferation by MSCs transfected with GFP or GADs. Where indicated, MSC were transfected with single or double amount of coding plasmids, or co-treated with IL-1β. Controls for T-cell proliferation include 1, basal, non-stimulated cultures; and 2, maximal stimulation (no MSC treatment). MSC doses in co-culture experiments were 50,000 (*3*,*8*,*13*,*18*), 40,000 (*4*,*9*,*14*,*19*), 30,000 (*5*,*10*,*15*,*20*), 20,000 (*6*,*11*,*16*,*21*) or 10,000 cells (*7*,*12*,*17*,*22*) and TCD4+ cells were kept constant at 300,000 cells per well. Similar immunosuppression was obtained using GADs expression or IL-1β priming. Image is a representative experiment out of 2 independent assays.

We next asked whether increased immunosuppression could be obtained using GAD transfected cells. If increased GABA levels were detected in MSC transfected with both GAD isoforms, we should therefore expect increased inhibition of T-cell proliferation when using GAD-67/GAD-65 co-transfected MSC. Immunosuppression assays were set using [1:30] (Fig 5A), [1:15] (Fig 5B) or [1:10] (Fig 5C) MSC:TCD4^+^ ratios, and T-cell proliferation was assessed by Flow Cytometry. In the absence of cytokine priming, GADs overexpression causes an increase in the immunosuppressive properties of MSC at ratios over 1:15. Notably, MSCs transfected only with GAD-67 do not show increased immunosuppressive function compared to control MSC in this experimental setting, at any ratio utilized. To further demonstrate this, dose-response experiments were performed, and MSCs primed with IL-1β were used alongside as a positive control to test for increased immunosuppression as described before. A dose response increase in immunosuppression was obtained using GAD-transfected cells compared to control and immunosuppression levels were comparable to those obtained with cytokine priming (Fig 5D). Our results demonstrate that maximal GABA secretion can be obtained by using GADs co-expression and that GADs overexpression enhances MSC immunosuppressive properties in the absence of priming.

### Increased Nitric Oxide levels in immunosuppression assays with MSC-GAD

**Fig 6:**
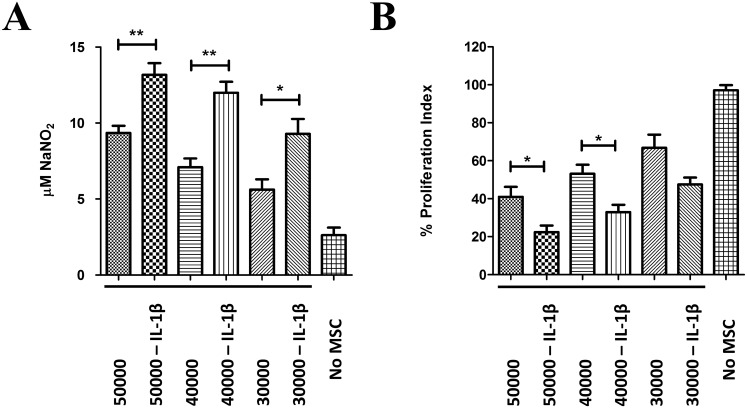
IL-1β priming increases NO levels and immunosuppressive properties of MSC. MSC were seeded and treated with IL-1β for 24 h as described before. The ending day for the cytokine treatment, total splenocytes were isolated from healthy C57BL/6 mice, labeled with CTV for proliferation assessment and stimulated with anti-CD3/anti-CD28 for 4 h. MSC were then added to pre-seeded splenocytes at indicated doses, and co-cultures were developed for additional 60 h. Then, conditioned media was recovered for nitric oxide determinations (A) and cells were resuspended for splenic T-cell proliferation assessment by FACS (B). Priming with IL-1β increased NO levels in cell culture and potentiates immunosuppressive properties of MSC. *, p<0.05; **, p<0.01 (Mann-Whitney *t*-test, N = 5).

**Fig 7:**
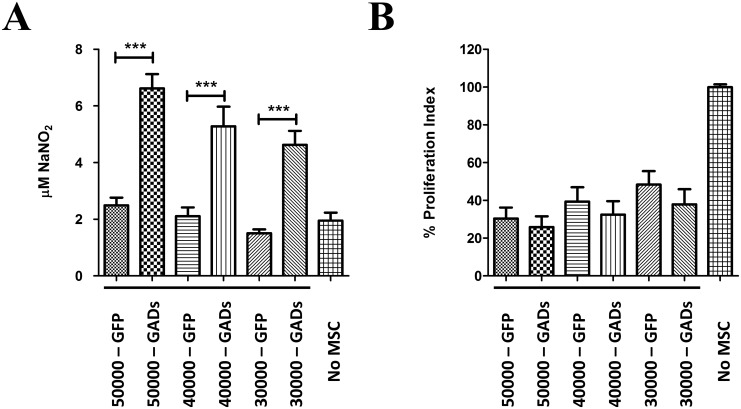
GADs expression increases NO levels and immunosuppressive properties of MSC. MSC were seeded, transfected with GADs or GFP, and recovered for 24 h as before. Total splenocytes were isolated from healthy C57BL/6 mice, and cells were labeled with CTV for proliferation assessment as described in Fig 6. MSC were then added to CTV-labeled splenocytes cells at indicated doses, and co-cultures were developed for additional 60 h. Then, conditioned media was recovered for nitric oxide determinations (A) cells were gently resuspended and splenic T-cell proliferation assessed by FACS (B). Expression of GADs caused an increase of NO levels in cell culture and potentiates immunosuppressive properties of MSC. *, p<0.05 (Mann-Whitney *t*-test, N = 4–8).

**Fig 8:**
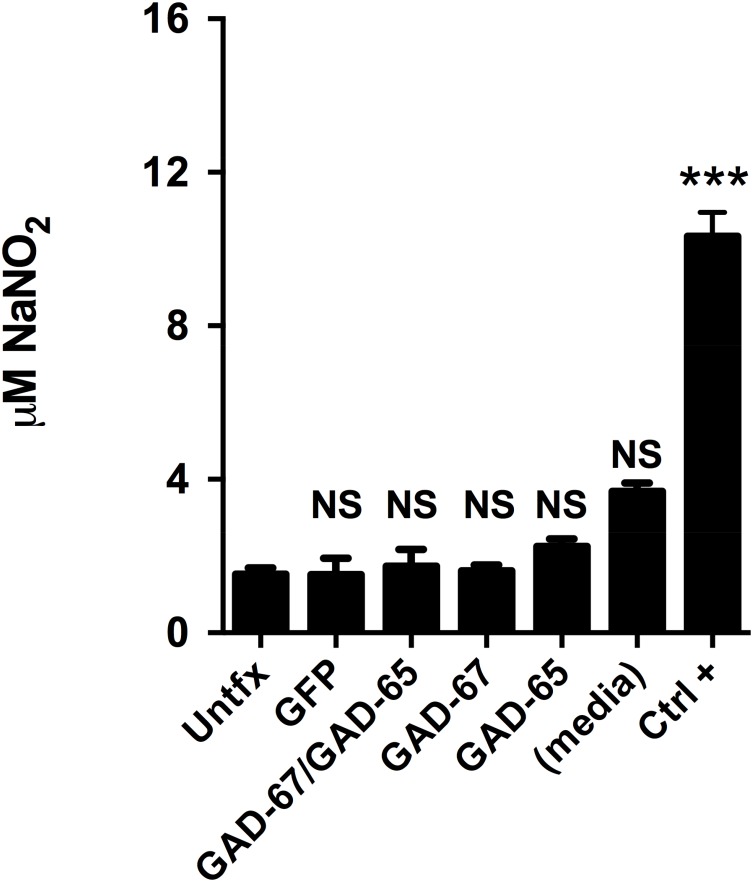
GADs expression does not increase nitric oxide levels *per se* in MSC. MSCs were seeded and transfected as indicated in Fig 4 or treated with cytokines as in S2 Fig. After 48 h, conditioned media was collected and assayed for NO levels as described in *Methods*. Plated media without cells was also included as control (media). GAD expression does not increase steady state levels of secreted nitric oxide. Results are from 4 independent experiments with determinations performed in triplicate. ***, p<0.001 (Kruskal-Wallis test with Dunn’s correction). NS, not statistically significant.

To gain insight into possible mechanism by which GADs-expressing MSC increase immunosuppressive properties, we analyzed secreted factors involved in MSC-mediated immunosuppression to evaluate possible interaction between immunosuppressive pathways. We focused on nitric oxide synthesis because GABA and NO signaling are linked in the setting of brain maturation, and given that both soluble mediators are potent immunosuppressors. We tested whether expression of GAD modifies nitric oxide production in co-culture experiments. Using co-cultures of activated splenocytes and GFP- or GAD-transfected MSC. As a positive control, we first determined the effect of IL-1β priming on MSC-induced immunosuppression and nitric oxide levels were measured by a fluorometric assay (DAN assay). To properly correlate NO levels and the degree of immunosuppression obtained, both nitric oxide levels and corresponding splenic T-cell proliferation were determined in the same well. A dose-dependent increase in nitric oxide levels was detected upon increasing doses of MSC (Fig 6A). A further statistically significant increase in nitric oxide levels was detected when using corresponding doses of MSC pre-stimulated with 20 ng/mL IL-1β for 24 h. Conversely, decreased T-cell proliferation was accordingly detected with increased doses of MSC and IL-1β priming stimulated these immunosuppressive properties, as expected (Fig 6B). Of note, a clear inverse correlation between the levels of nitric oxide and splenic T-cell proliferation was observed. Measurement of nitric oxide by the DAN assay relies on the acidic reaction of DAN with the nitrosating agent dinitrotrioxide generated from acidified nitrite or from the autooxidation of NO [64,65]. Therefore, changes measured by the DAN assay may also be the result of changes in nitrate reductase or other cellular sources of nitrite in the co-culture as well. To rule out this possibility, we performed similar experiments and determined iNOS protein levels in whole co-culture lysates. It has been previously determined that iNOS is mostly expressed by MSC and is upregulated upon co-culture with stimulated splenocytes or T-cells, which is further increased by cytokine priming [24]. We confirmed increased iNOS protein levels in those conditions where increased nitric oxide levels by the DAN assay was detected (S4 Fig), validating our technique and confirming previous reports [20,24]. Therefore we have a suitable assay to test immunosuppressive properties of MSC on splenic T-cell responses via changes in nitric oxide levels produced via iNOS induction. To determine whether GAD-expressing cells behaved similar to licensed MSC, we transfected cells with GFP or GADs. Cells were kept for 24 h in the absence of cytokine licensing and immunosuppression assays were performed using increasing doses of MSC as before. GFP expression had little if any effect on nitric oxide production in co-culture experiments using unlicensed MSCs. Surprisingly, a strong dose-dependent rise in nitric oxide levels was detected when using MSCs that were transfected with GADs (Fig 7A). Importantly, increased levels of iNOS protein were also detected in the immunosuppression assays that used GAD-expressing MSC, compared to control cells (S5 Fig). A slight but consistent increase in MSC-mediated immunosuppression was also detected for all doses analyzed (Fig 7B). To corroborate GAD expression in our co-culture experiments, we measured secreted GABA levels in the conditioned media at end point as an indirect readout to demonstrate GAD expression (S6 Fig). Dose-dependently increased GABA levels were detected in immunosuppression conditions that used MSC-GABA. It is well known that activated T-cells trigger the induction of inducible nitric oxide synthase via IFN-γ-mediated mechanisms to produce nitric oxide-dependent immunosuppression in murine MSCs [20,24]. The strong effect GAD expression has on nitric oxide levels in the setting of immunosuppression could be the result of direct effects of GABA on MSC. To rule out this possibility, we determined secreted NO levels in cultures of control- or GAD-transfected MSC alone, in the absence of co-cultured splenocytes. Neither GAD expression, nor transfection of GAD-67 or GAD-65 isoform alone, nor GFP expression modified levels of secreted nitric oxide (Fig 8), indicating that GAD expression *per se* does not change the production of this immunosuppressive agent in MSCs. Therefore, GABAergic activation of MSC increases NO levels in a T-cell or splenocytes-dependent manner, while GABA secretion does not rely on the presence of immune cells. Collectively, our results indicate that GABAergic activation of MSC by GAD expression increases both nitric oxide production and secreted GABA levels, two potent immunosuppressive agents that may be responsible for the increased immunosuppression observed with MSC-GAD.

## Discussion

We demonstrated that pro-inflammatory cytokines selectively regulate GAD-67 expression in murine BMMSCs. IL-1β increased mRNA levels but IFN-γ was required to increase GAD-67 protein expression. Gain of function experiments demonstrate that overexpression of GAD-67 together with GAD-65 significantly increases secreted GABA levels and MSC-mediated immunosuppression in the absence of any pro-inflammatory licensing. The increased immunosuppression capabilities of MSC by GAD expression occur in the setting of splenic or isolated TCD4+ cell proliferation. Importantly, GAD expression evokes an increase in both secreted GABA and nitric oxide levels in MSC co-cultured with splenocytes, and this effect may require MSC-to-immune cells communication, as this effect was not observed in pure cultures of MSC expressing GAD. These results collectively demonstrate that active GABA secretion by MSC increases MSC-mediated immunosuppression and suggests that crosstalk between GABA and nitric oxide signaling in the setting of MSC-mediated immunosuppression occurs.

### GABA secretion by MSC and its role in MSC-induced immunosuppression

Gene expression regulation of GABA synthesis and GABA degradation machineries has never been addressed in undifferentiated MSC. We readily detected transcripts for GABA transaminase (ABAT) and GAD-67 in basal conditions (Fig 3A), and GAD-67 protein was successfully detected in MSC (Fig 3B). Moreover, secreted GABA levels were detected in the low μM range in unlicensed state (see Fig 4B and text therein), indicating that basal production and secretion of GABA do occur in MSC. Expression of GAD-67 was selective, and complete absence of GAD-65 expression by RT-qPCR and Western blot is intriguing. In neurons, GAD-65 expression is required for efficient packaging and secretion of GABA into synaptic vesicles in the central nervous system [86,92]. Knockout animal data however indicate that GAD-67 mediates the bulk of GABA synthesis in the brain [83,84]. Reconciling observations now indicate that GAD-67 and GAD-65 form stable heterodimers in isolated membranes from both brain and cultured neurons [89,90,93] and it has been proposed that functional heterodimers of GAD-67 and GAD-65 might be important for GABA secretion at GABAergic synapses [94]. Of note, a GAD-65 independent mechanism for GAD-67 membrane anchoring also occurs in synaptic membranes [90,91,95]. We observed that both independently expressed GAD-65 or GAD-67 increased GABA secretion in MSC compared to GFP or untransfected cells (Fig 4). However, GAD-67/GAD-65 co-expression evoked an even higher increase in GABA secretion in MSC, even though total equal amounts of plasmids for GAD were transfected and proteins levels for GAD expressed accordingly, suggesting that functional GAD heterodimers are also formed in MSC (Fig 4). However, other mechanisms of GABA secretion cannot be ruled out. In non-neuronal cells GABA secretion via reversal of GABA transporters GAT-1 and GAT-2, has been described for astrocytes and neurons [96,97]. Whether MSC do express functional GAT transporters is not known. Additional experiments to better understand the mechanism of endogenous GABA secretion by undifferentiated MSC and the role of GAD isoforms and GAT transporters are needed.

Regardless mechanisms of GABA secretion in GAD-transfected MSC, increased immunosuppression and GABA secretion was observed for doubly transfected cells compared to GFP controls. Noteworthy, this effect was observed both using purified CD4+ T-cells or splenocytes (Fig 5). This is particularly important, as possible undesired effects might have been observed when switching from TCD4+ to splenocytes. It has been previously reported that macrophages induce a pro-inflammatory response to GABA via uptake of L-glutamine/GABA and subsequent GABA-to-succinate conversion, where succinimidilation of proteins causes activation of HIF-1a signaling pathway and reprogramming of innate immunity cells to a pro-inflammatory phenotype [98]. We were successful to induce immunosuppression in the setting of splenocytes, indicating that suppressive function dominates when using MSC-GAD. Unexpected results, however, were obtained when using GAD-67 only expression. Although we described that pro-inflammatory cytokines induced endogenous GAD-67 expression, and that increased GABA levels were observed in GAD-67 transfected pure MSC cultures (Fig 4), overexpression of GAD-67 alone was not sufficient to increase immunosuppressive properties of MSC (Fig 5A–5C). As co-transfection resulted in the higher amount of GABA secreted compared to single GAD-65 or GAD-67 expression (Fig 4), we believe that threshold levels for GABA are reached in order for immunosuppression to occur. In this regard, a recent report indicates that IFN-γ stimulation of human macrophage lineage cells decreases extracellular GABA content by induction of GAT transporters [99]. It would be most interesting to analyze the contribution of pro-inflammatory cytokines to GABA secretion in GAD-67, GAD-65 or GAD-67/GAD-65 transfected MSC to better understand how an inflammatory milieu could affect GABA secretion, and to further correlate it to the immunosuppressive capabilities of MSC as well. Similarly, we are planning side-directed mutagenesis experiments on GAD-65 to impair membrane association of GAD-65 to cell membranes [100] to provide proof of concept of heterodimer formation in co-transfected MSC and to demonstrate secreted GABA levels threshold reach in immunosuppression experiments.

### Gene expression regulation of GAD-67 by pro-inflammatory cytokines

In isolated MSC cultures, cytokine treatment increased GAD-67 expression levels selectively, because GAD-65 mRNA and protein levels were undetected in any of the conditions tested (Fig 3A and 3B). Interestingly, complex gene expression regulation was observed for GAD-67. Although IL-1β raised GAD-67 mRNA levels (Fig 3A), IFN-γ was important to increase GAD-67 steady state protein levels (Fig 3B). It is worth mentioning that both regulation of proteasomal activity and miRNA induction by IFN-γ have been described, and similar mechanisms may underlie IFN-γ effects on GAD-67 protein expression [101,102]. The functional role for endogenous GAD-67 expression and regulation by pro-inflammatory cytokines remains elusive. GAD-67 overexpression alone don´t make MSC cells more immunosuppressive (Fig 5A–5C). Results from GAD co-expression experiments and immunosuppressive properties of MSC indicate that expression of GAD-65 is required in order GAD-67 to show a positive effect. We speculate that GAD-65/GAD-67 co-expression may escape the deleterious effects of IFN-γ, possibly because of a proper trafficking of GAD-67 for GABA secretion when co-expressed to GAD-65. However, loss of function experiments are required to clearly establish a functional role for GAD-67 expression in the immunosuppressive properties of MSC.

### IL-1β priming suffices to prime Invitrogen MSC

Our results indicate that Invitrogen bone-marrow derived MSC is a qualified and trustable source of MSC for *in vitro* immunosuppression studies. Interestingly, we uncovered a new type of cytokine licensing, namely induction of anti-inflammatory properties of MSC via IL-1β priming in this heterogeneous cell preparation. Most reports on MSC licensing rely on Dr. Shi findings with murine MSC that demonstrate requirement for combinatorial treatment of MSC with IFN-γ and either TNF-α or IL-1 [20,21]. We have two possible explanations for our discrepancy in cytokine requirement for cell licensing. First, Dr. Shi experiments were performed with a clonal population of MSCs, suggesting that properties might be attributable to the clonal effects, and MSC preparation may overcome the need for the combination of two cytokines because enough IFN-γ receptor levels in the heterogeneous population. Interestingly, differential IFN-γ receptor levels have been described for human MSC that affect their immunosuppressive capabilities [103] and priming with IL-1β alone stimulates immunosuppression capabilities of human MSC [104]. Second, cultures of proliferating T-cell are expected to continuously produce IFN-γ [105], so IL-1β priming might render MSC loaded with immunosuppressive mRNAs as a “first signal” for a later encounter with IFN-γ in the co-culture to terminate the induction of effector genes required for immunosuppression, like COX-2, iNOS, and GAD-67. Regardless priming details, Invitrogen MSC are suitable for immunosuppression assays and show normal functions, including inhibition of T-cell proliferation (Figs 1, 2, 5, 7 and 8), induction of nitric oxide synthesis by pro-inflammatory cytokines (S2 Fig), and dose dependent stimulation of immunosuppressive properties and nitric oxide secretion in co-cultures with activated splenocytes (Fig 7).

### Increased nitric oxide production by GAD expression in MSC

Increased secreted nitric oxide levels and cellular iNOS protein were detected in GAD-transfected MSC co-cultures in immunosuppression assays using splenic polyclonal T-cell responses. Increase was dose-dependent to the dose of MSC added and didn´t occur when using GFP transfected MSC (Fig 7A, S5 Fig). More importantly, increase in nitric oxide levels does not occur when using pure GAD-transfected MSC cultures (Fig 6), suggesting that cell-to-cell contact or soluble factors by activated splenocytes are required for nitric oxide levels increase in the conditioned media of co-cultures using GAD-expressing MSC. Interplay of this two signaling pathways have long been suggested in both neuronal and peripheral tissues [106,107,108]. Because nitric oxide production in the setting of MSC-mediated immunosuppression rely on iNOS gene expression in MSC [24], we hypothesize that an autocrine feedback loop for nitric oxide production in GABA-producing MSC cells occurs in the setting of interaction with activated splenocytes. It will be of great interest to study the regulation of iNOS gene expression by pro-inflammatory cytokines in GAD-manipulated MSC or GABA treated cells to further dissect this regulation. Although direct evidence showing GABAAR modulation of IFN-γ receptor signaling is lacking, regulation in the opposite direction do exist. It has been demonstrated that NO affects GABA secretion and GABAAR function by both cGMP-dependent signaling and nitrosilative mechanisms [109]. Nonetheless, further experiments are required to identify particular mediators, such as specific combinations of secreted cytokines or cell-to-cell contact molecules on immune cells, which can contribute to iNOS expression regulation in GAD-transfected MSC. Additionally, the use of specific inhibitors targeting NO and GABA production will be required to evaluate their respective contributions, and to better depict a possible crosstalk of NO and GABA signaling in MSC-mediated immunosuppression using GAD-transfected cells.

## Conclusion

Our results demonstrate for the first time that endogenous GABA synthesis machinery is upregulated by pro-inflammatory cytokines in MSC cells. Importantly, manipulation of GABAergic signaling in MSC increases their *in vitro* immunosuppressive properties in the absence of pro-inflammatory pre-stimulation by mechanisms that may involve both increased GABA secretion and nitric oxide production. We have unveiled a previously unreported effect in the expression of iNOS protein and nitric oxide production by the solely modification of levels for GAD-67 and GAD-65 glutamate decarboxylases in MSC. Importantly, this regulation only occurs in the context of its interaction with immune cells, indicating that both GABA and NO pathways are interrelated in the setting of immune regulation. Since GABA and NO pathways regulate important immunosurveillance activities, and given that both molecules are able to directly inhibit T-cell proliferation [24, 40, 47, 50, 110, 111], these findings may open a new avenue for basic and translational research aiming at controlling deregulated T-cell proliferation in disorders of the immune system by using MSC with modified GAD content. It may be of great interest to extrapolate these findings to human MSC, and to evaluate efficacy of GAD-MSC therapy in autoimmune preclinical models like EAE and CIA.

## Supporting Information

S1 Fig Twenty-four hours pre-treatment with IL-1β also increases immunosuppressive properties of MSC. MSC were seeded, treated with cytokines and tripsinized for TCD4+ co-cultures setting exactly as in Fig 2, but cells were grew 48 h before adding cytokines, and cells were incubated only 24 h with cytokines. 24 h priming with IL-1β also increases immunosuppressive properties of MSC. *, p<0.05 (Kruskal-Wallis test with Dunn’s correction, N = 3).
(TIF)

S2 Fig Corroboration of IFN-γ signaling activation in MSCs. MSC were seeded at 4,800 cells/cm^2^ and grew for 24 h. MSC cultures were then supplemented with 20 ng/mL IL-1β, 25 ng/mL IFN-γ, or the combination of both cytokines as described in *Methods*, and cells were grew for additional 24 or 48 h. (A) Cells were lysed and iNOS protein levels were determined by Western blot. β-actin was used as a loading control and the iNOS/β-actin ratio was quantitated by densitometric analysis (*Right panel*). (B) Conditioned media was cleared and NO levels measured as nitrites, using a fluorescence-based assay. Combined cytokine priming increases both cellular iNOS protein levels and secreted nitric oxide after both 24 and 48 h treatment. *, p<0.05, **, p<0.01, ***, p<0.001 (Kruskal-Wallis test with Dunn’s correction).
(TIF)

S3 Fig GAD-65 is not induced by IL-1β and IFN-γ in MSCs. MSC were seeded, treated and processed as in Fig 3, and 30 μg protein were sampled for determination of GAD-65 levels by Western blot. GAD-65 was not detected in any of the experimental conditions. 15 μg of total brain lysate was used as a positive control (+).
(TIF)

S4 Fig Elevation of iNOS protein expression by IL-1β priming of MSC in the setting of immunosuppression assays. Experiments were performed identically as in Fig 6 and cell lysates were prepared as described in *Methods*. (A) iNOS and β-actin protein levels were determined by Western blot and the iNOS/β-actin ratio quantitated by densitometric analysis. (B) A representative Western blot utilized for densitometric quantitation showing the expected band sizes for iNOS (~120 kDa) and β-actin (~45 kDa). (C) Nitrite levels, and (D) T-cell proliferation assessment in sister wells for the co-culture assays. Corresponding increased iNOS protein levels; nitrites levels measured by DAN and decreased T-cell proliferation demonstrate enhanced immunosuppression via nitric oxide production upon IL-1β priming. *, p<0.05 (Kruskal-Wallis test with Dunn’s correction); #, p<0.05 (Mann Whitney t-test); N = 4.
(TIF)

S5 Fig Elevation of iNOS protein expression in the setting of immunosuppression assays using GAD-transfected MSC. Experiments were performed identically as in Fig 7 and cell lysates were prepared as described in *Methods*. (A) iNOS and β-actin protein levels were determined by Western blot and the iNOS/β-actin ratio quantitated by densitometric analysis. (B) Representative Western blot results showing expected size bands of iNOS (~120 kDa) and β-actin (~45 kDa) used for quantitation. Matching nitrite levels, (C), and T-cell proliferation indexes, (D), demonstrate GAD transfection increases nitric oxide production and their immunosuppressive properties in co-culture. *, p<0.05 (Kruskal-Wallis test with Dunn’s correction, N = 3).
(TIF)

S6 Fig Detection of GABA in the setting of immunosuppression assays. Dose-dependent inhibition of splenic T-cell responses (anti-CD3/anti-CD28) were performed using MSCs treated with IL-1β or transfected with GADs. Non-treated cells or GFP transfected cells were used as respective MSC controls. Conditioned media was processed as described in *Methods* and GABA levels measured as before. A dose-dependent increase in GABA levels was only detected in conditioned media from activated splenocytes co-cultured with GAD-transfected cells, compared to stimulated splenocytes without addition of MSC, (+). Splenocytes with no stimulation were also included as a control, (-). *, p<0.05, **, p<0.01 (Kruskal-Wallis test with Dunn’s correction, N = 3).
(TIF)

S1 Protocol Indirect determination of nitric oxide with 2,3-diaminonapthalene (nitrite detection). (PDF)
