# Supplementary figures and images for: Overexpression of Glutamate Decarboxylase in Mesenchymal Stem Cells Enhances Their Immunosuppressive Properties and Increases GABA and Nitric Oxide Levels
Source: PLoS One. 2016 Sep 23;11(9):e0163735. doi: 10.1371/journal.pone.0163735 (PMC5035029; doi:10.1371/journal.pone.0163735)

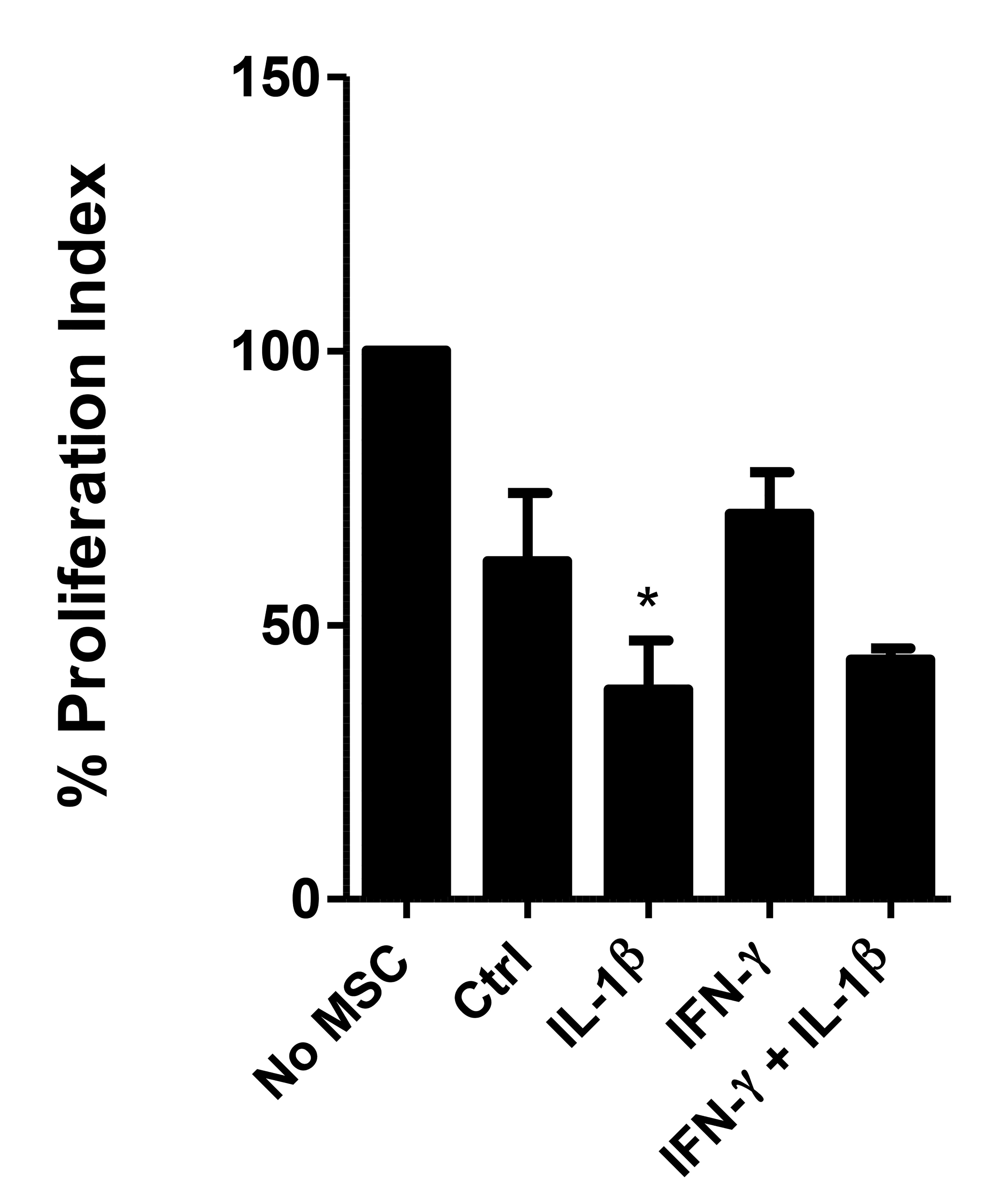

Supplement: S1 Fig — MSC were seeded, treated with cytokines and tripsinized for TCD4+ co-cultures setting exactly as in Fig 2, but cells were grew 48 h before adding cytokines, and cells were incubated only 24 h with cytokines. 24 h priming with IL-1β also increases immunosuppressive properties of MSC. *, p<0.05 (Kruskal-Wallis test with Dunn’s correction, N = 3). (TIF) [file pone.0163735.s001.tif]

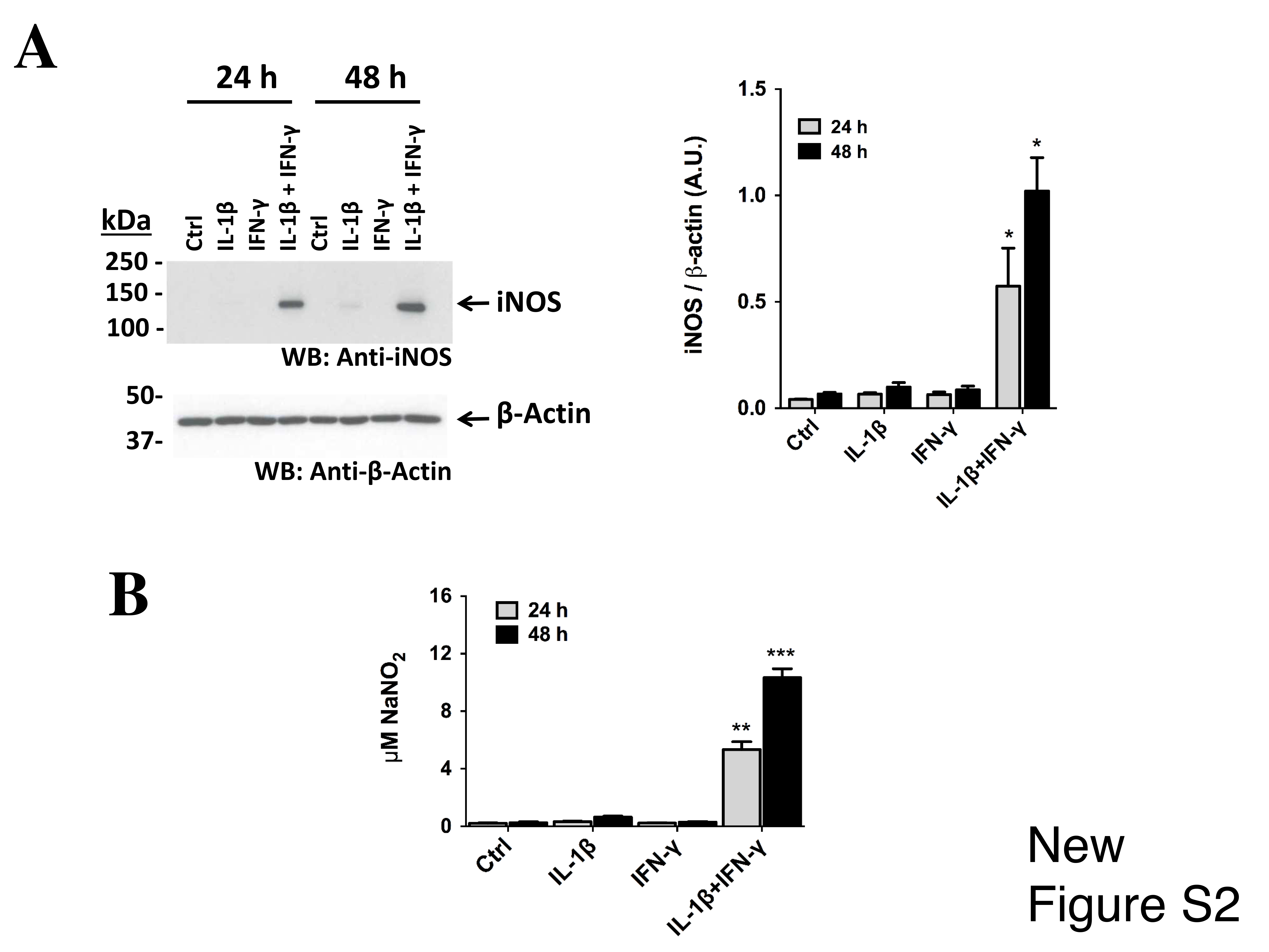

Supplement: S2 Fig — MSC were seeded at 4,800 cells/cm2 and grew for 24 h. MSC cultures were then supplemented with 20 ng/mL IL-1β, 25 ng/mL IFN-γ, or the combination of both cytokines as described in Methods, and cells were grew for additional 24 or 48 h. (A) Cells were lysed and iNOS protein levels were determined by Western blot. β-actin was used as a loading control and the iNOS/β-actin ratio was quantitated by densitometric analysis (Right panel). (B) Conditioned media was cleared and NO levels measured as nitrites, using a fluorescence-based assay. Combined cytokine priming increases both cellular iNOS protein levels and secreted nitric oxide after both 24 and 48 h treatment. *, p<0.05, **, p<0.01, ***, p<0.001 (Kruskal-Wallis test with Dunn’s correction). (TIF) [file pone.0163735.s002.tif]

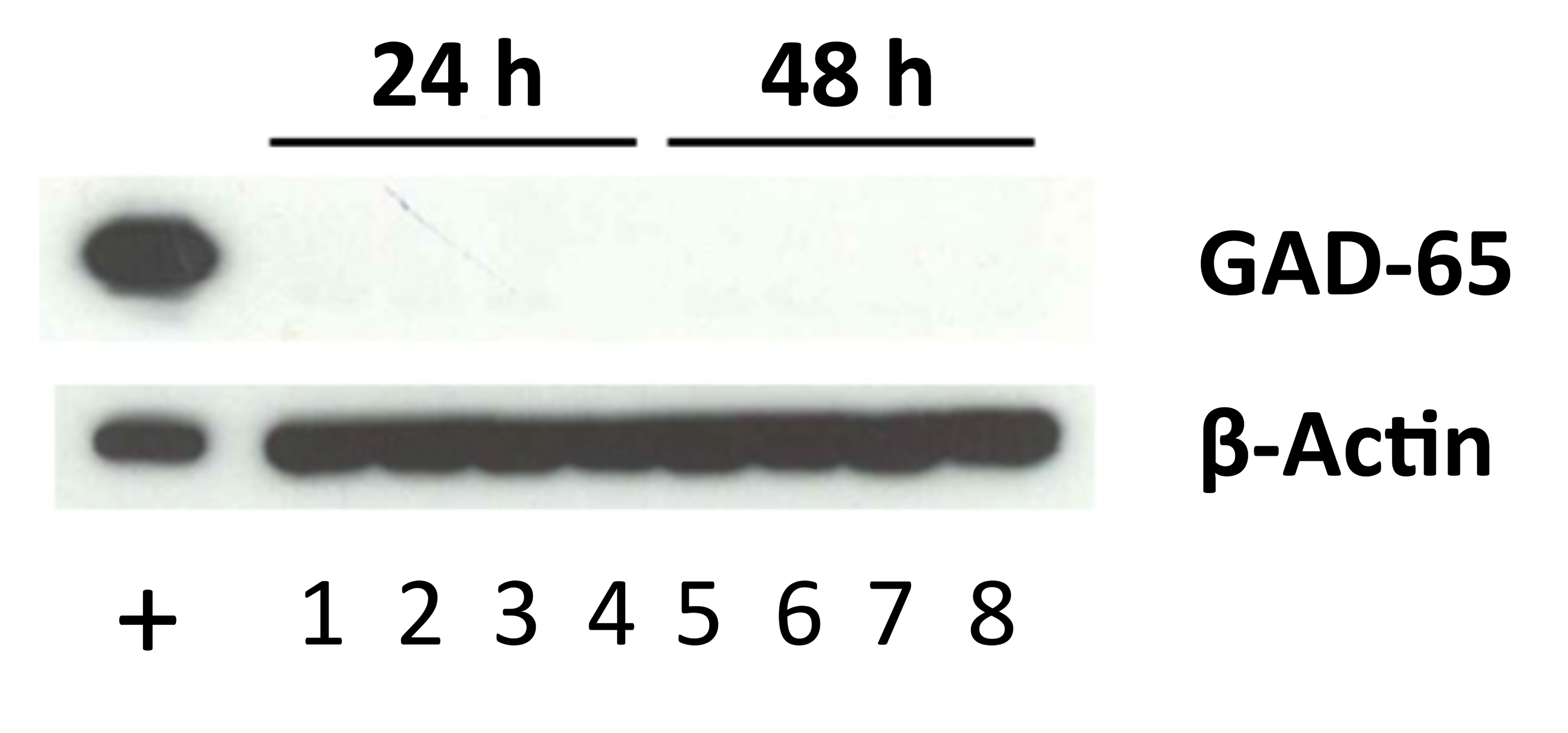

Supplement: S3 Fig — MSC were seeded, treated and processed as in Fig 3, and 30 μg protein were sampled for determination of GAD-65 levels by Western blot. GAD-65 was not detected in any of the experimental conditions. 15 μg of total brain lysate was used as a positive control (+). (TIF) [file pone.0163735.s003.tif]

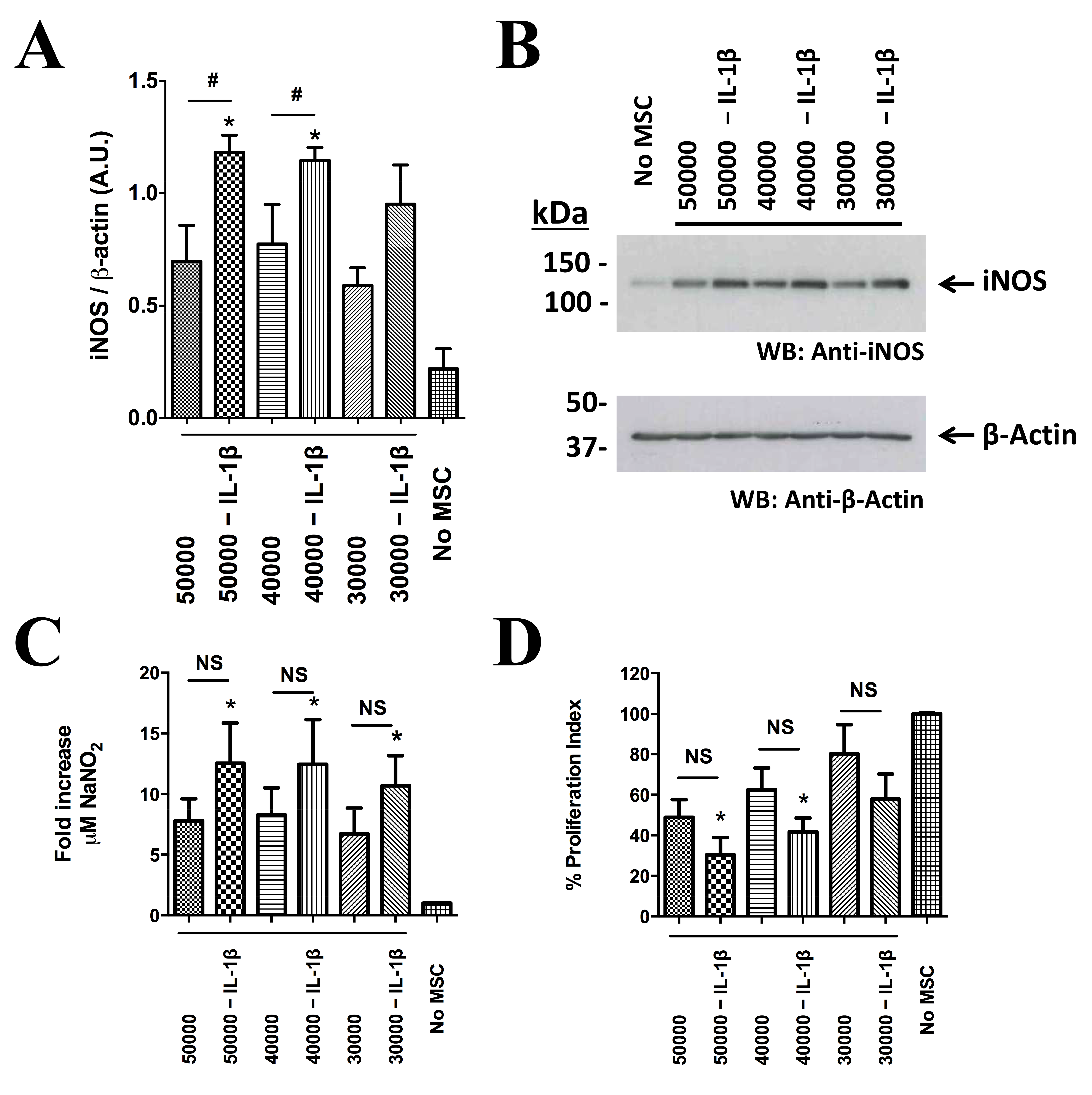

Supplement: S4 Fig — Experiments were performed identically as in Fig 6 and cell lysates were prepared as described in Methods. (A) iNOS and β-actin protein levels were determined by Western blot and the iNOS/β-actin ratio quantitated by densitometric analysis. (B) A representative Western blot utilized for densitometric quantitation showing the expected band sizes for iNOS (~120 kDa) and β-actin (~45 kDa). (C) Nitrite levels, and (D) T-cell proliferation assessment in sister wells for the co-culture assays. Corresponding increased iNOS protein levels; nitrites levels measured by DAN and decreased T-cell proliferation demonstrate enhanced immunosuppression via nitric oxide production upon IL-1β priming. *, p<0.05 (Kruskal-Wallis test with Dunn’s correction); #, p<0.05 (Mann Whitney t-test); N = 4. (TIF) [file pone.0163735.s004.tif]

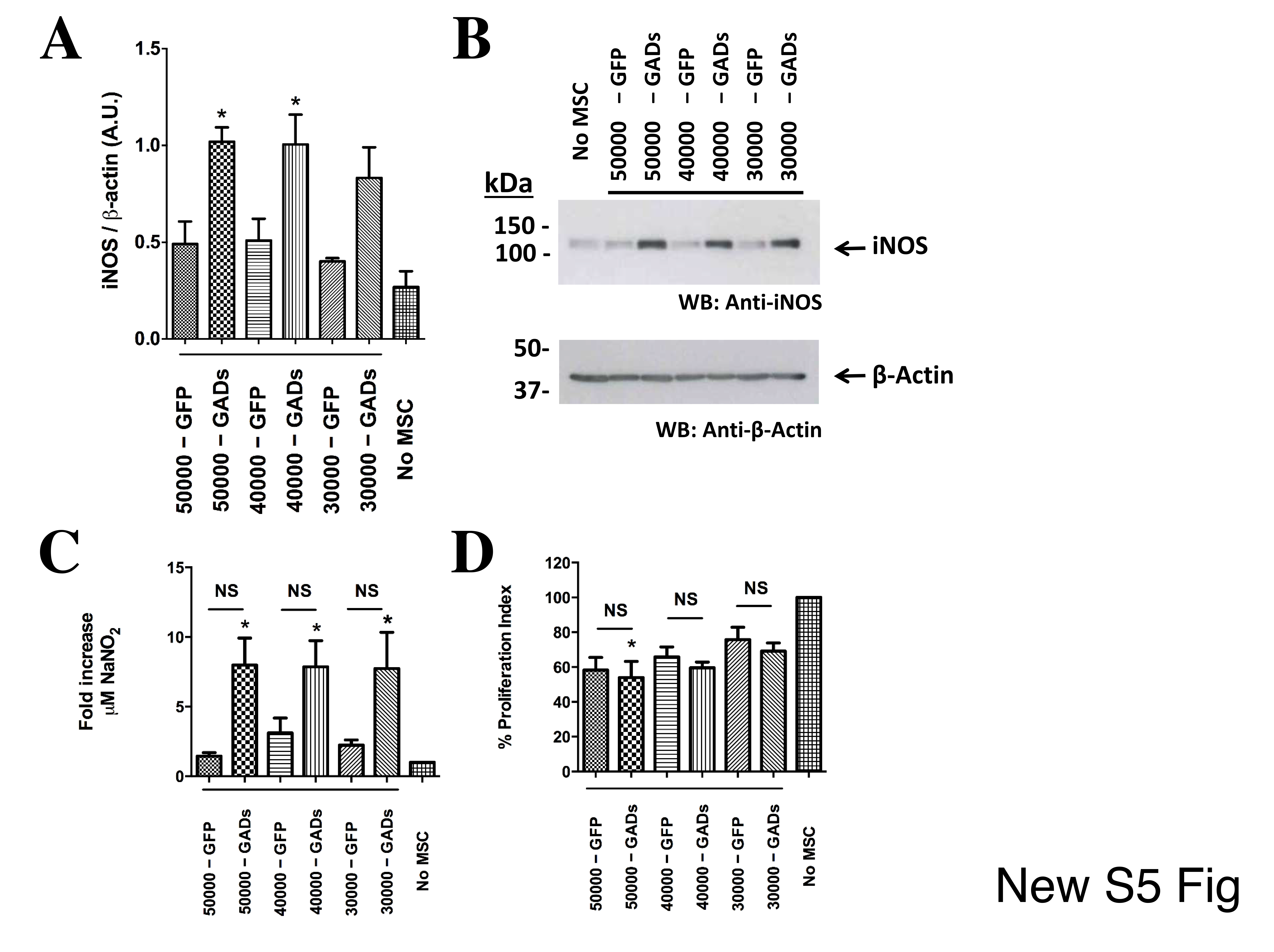

Supplement: S5 Fig — Experiments were performed identically as in Fig 7 and cell lysates were prepared as described in Methods. (A) iNOS and β-actin protein levels were determined by Western blot and the iNOS/β-actin ratio quantitated by densitometric analysis. (B) Representative Western blot results showing expected size bands of iNOS (~120 kDa) and β-actin (~45 kDa) used for quantitation. Matching nitrite levels, (C), and T-cell proliferation indexes, (D), demonstrate GAD transfection increases nitric oxide production and their immunosuppressive properties in co-culture. *, p<0.05 (Kruskal-Wallis test with Dunn’s correction, N = 3). (TIF) [file pone.0163735.s005.tif]

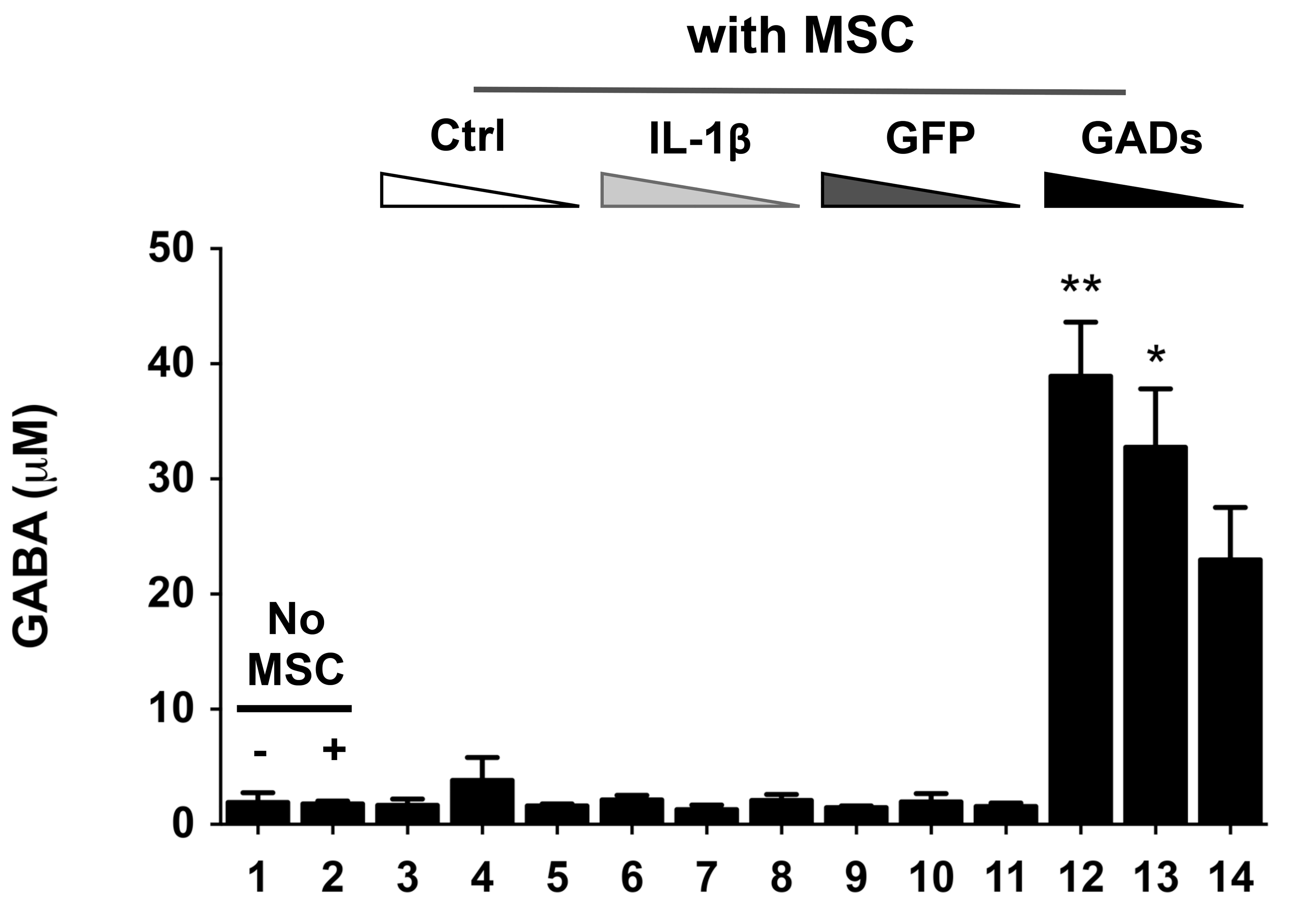

Supplement: S6 Fig — Dose-dependent inhibition of splenic T-cell responses (anti-CD3/anti-CD28) were performed using MSCs treated with IL-1β or transfected with GADs. Non-treated cells or GFP transfected cells were used as respective MSC controls. Conditioned media was processed as described in Methods and GABA levels measured as before. A dose-dependent increase in GABA levels was only detected in conditioned media from activated splenocytes co-cultured with GAD-transfected cells, compared to stimulated splenocytes without addition of MSC, (+). Splenocytes with no stimulation were also included as a control, (-). *, p<0.05, **, p<0.01 (Kruskal-Wallis test with Dunn’s correction, N = 3). (TIF) [file pone.0163735.s006.tif]
