# Supplementary material for: Overexpression of Glutamate Decarboxylase in Mesenchymal Stem Cells Enhances Their Immunosuppressive Properties and Increases GABA and Nitric Oxide Levels
Source: PLoS One. 2016 Sep 23;11(9):e0163735. doi: 10.1371/journal.pone.0163735 (PMC5035029; doi:10.1371/journal.pone.0163735)

Nitric oxide determination with 2,3-diaminonapthalene (nitrite detection)

Adapted from **Misko et al, 1993** (A fluorometric assay for the measurement of nitrite in biological samples (1993) Anal Biochem, vol 214, No 1, pp 11-16) and **Kleinhenz et al, 2003** (Detection of endothelial nitric oxide release with the 2,3-diaminonapthalene assay (2003) Free Radical Biology & Medicine, vol 34, No 7, pp 856-861). DAN stock concentration taken from **Fang et al, 2009** (Fluorometric determination of nitrite with 2,3-diaminonaphthalene by reverse phase HPLC under alkaline conditions (2009), J Pharmacol Tox Met, vol 59, No 3, pp 153-155).

DAN stock

- 2 mg/mL (MW= 158.2 g/mol) in HCl 0.62 N
- 2.5 mL HCl 0.62 N => 129.2 µL fuming Hydrochloric acid + 2370.8 µL MiliQ water
- Weigh 2 mg, bring to 2 mg/mL and store in 25 µL aliquots at -20°C. Protect from light.

$NaNO_2$ stock

- First, make 200 mM (MW= 69 g/mol) in MiliQ water
- Weight 0.138 g, bring to 200 mM by adding 10 mL MiliQ water
- Then make a 1:10 dilution (1 mL 200 mM $NaNO_2$ + 9 mL MiliQ water) and store frozen as 1 mL aliquots (20 mM) at -20°C. Protect from light.

PROTOCOL

(Amounts indicated are good enough for 1 full 96-well plate = 11 points standard curve, 21 samples, all in triplicate)

Be sure to have at least 6 mL matching media for preparation of the standard curve before starting. All samples have to be pre-clarified by centrifugation 10 min 400 *xg*, and brought to RT, protected from light.

1. Thaw 1 aliquot of the 2 mg/mL DAN stock at RT
2. Equilibrate 1 aliquot of 20 mM $NaNO_2$ stock at RT. The thawed aliquot can be stored protected from light in the fridge for later use (<1 month).
3. Prepare 2.5 mL fresh 0.62 N HCl
4. In a 5 mL polypropylene tube dispense 25 µL DAN + 1,975 µL 0.62 N HCl
5. Label Eppendorf tubes 1-to-10 and "1:100"
6. Prepare 1:100 $NaNO_2$ in the same media samples will be
   (2 µL $NaNO_2$ 20 mM + 198 µL matching media, to make 200 µL 200 µM)

7. Prepare the standard curve in the same media samples will be:
   - **Prepare Tubes 2-10**: Place 500 µL matching media
   - **Tube 1**: Place 100 µL 200 µM $NaNO_2$, add 900 µL matching media. Vortex for 15 seconds.
   - **Tubes 2-10**: take 500 µL from the previous dilution and mix with the already placed 500 µL media by pipetting. Vortex for 15 seconds the two-fold dilution before continuing with the next point of the serial dilution.

   All standard curve samples will have 500 µL, excepting for the last one that will have 1 mL.

8. In triplicate, in a black plate (Costar 3915), dispense 100 µL of the standard curve tubes 1-10, the background media and the samples
9. Put the diluted 0.025 mg/ml DAN solution in a small basin
10. Add 20 µL DAN 0.025 mg/mL to each sample using a multichannel pipette. Mix by pipetting up and down 6 times.
11. Gently tap the multiwell plate to mix the contents.
12. Incubate 15 min at RT in the dark with no mixing.
13. Add 20 µL NaOH 0.7 N per well using a multichannel pipette. Mix by pipetting up and down 6 times.
14. Gently tap the multiwell plate to mix the contents.
15. Read in the fluorescence plate reader

Use the following settings for an INFINITE M-1000 Pro plate reader:

Shaking (Linear) Duration:	5	s
Shaking (Linear) Amplitude:	2	mm
Shaking (Linear) Frequency:	654	rpm

Label: DAN		
Mode	Fluorescence Top Reading	
Multiple Reads per Well (Circle (filled))	3 x 3	
Multiple Reads per Well (Border)	500	µm
Excitation Wavelength	365	nm
Emission Wavelength	410	nm
Excitation Bandwidth	10	nm
Emission Bandwidth	20	nm
Gain	50-to-70	Manual
Number of Flashes	50	
Flash Frequency	400	Hz
Integration Time	20	µs
Lag Time	0	µs
Settle Time	10	ms
Z-Position (Manual)	20000	µm

Metrics of Standard curves prepared in different culture medias, and examples of raw fluorescence data.

Co-culture media

	metrics of DAN assay (standard curve) co-culture					
**Fecha Exp.**	**σ**	**Interseccion**	**S (slope)**	**LOD**	**LOQ**	**R2**
**20150717**	24.05246003	-67.15385863	609.998139	0.118291148	0.394303826	0.9981
**20150829**	15.82192572	-71.17471956	765.7028251	0.061989816	0.20663272	0.9904
**20150831**	12.42309677	-118.5295071	553.8032585	0.067296986	0.224323288	0.9959
**20150928**	22.5166605	-13.72341115	684.1784878	0.098731519	0.329105064	0.9953
**20151005**	39.17056718	194.9102261	473.9519539	0.247940114	0.826467047	0.9954
**20151009**	11.53256259	-46.34893005	351.6473047	0.098387468	0.327958225	0.9989
**20151019**	8.598691761	-75.71133268	647.6684104	0.039829139	0.132763797	0.9989
**20151031**	3.785938897	-18.9688716	276.1419987	0.041130349	0.137101162	0.9995
**20151102**	7.094598885	-10.92996109	247.2179968	0.086093233	0.286977444	0.9995
**20160613**	25.42308662	-210.0106299	988.551373	0.077152551	0.257175169	0.9958
**20160620**	5.859465277	-103.4839159	904.8045772	0.019427837	0.064759457	0.9997
**20160627**	106.3249735	-31.32910922	1731.256291	0.184244772	0.61414924	0.9987
**20160704**	116.0116134	540.8723174	1710.624675	0.203454823	0.678182743	0.9837
			ave	0.103	0.345	0.996
			sem	0.019	0.064	0.001

MSC media

	metrics of DAN assay (standard curve) pure MSC culture					
**Fecha Exp.**	**σ**	**Interseccion**	**S (slope)**	**LOD**	**LOQ**	**R2**
**20160615**	2	-35.64758854	367.5541701	0.016324124	0.054413748	0.9983
**20160622**	39.85304444	7.111788052	1203.359709	0.099354443	0.331181476	0.9919
**20160706**	85.36800905	20.47268254	2161.209052	0.118500349	0.395001164	0.9994
**20160707**	35.00476158	-300.6595182	2224.186937	0.047214685	0.157382282	0.9948
**20160709**	63.31139971	-17.59699206	2330.249883	0.081508082	0.271693608	0.9986
**20160721**	104.517941	-291.7151279	1598.723369	0.196127629	0.653758762	0.9892
**20160827**	90.87665634	-363.879845	1917.233927	0.142199637	0.473998791	0.9947
			ave	0.100	0.334	0.995
			sem	0.023	0.075	0.001

Co-culture media

µM NaNO2	i	ii	iii		i	ii	iii	ave	sd	cv				
20	21914	21281	20886		20670.67	20037.67	19642.67	20117	518.5714	2.577777				
10	10010	10274	10079		8766.667	9030.667	8835.667	8877.667	136.9197	1.542294				
5	5352	5332	5466		4108.667	4088.667	4222.667	4140	72.28647	1.74605				
2.5	3280	3517	3294		2036.667	2273.667	2050.667	2120.333	132.9749	6.271417				
1.25	2259	2220	2274		1015.667	976.6667	1030.667	1007.667	27.87472	2.766264				
0.625	1751	1767	1792		507.6667	523.6667	548.6667	526.6667	20.66398	3.92354				
0.3125	1562	1497	1545		318.6667	253.6667	301.6667	291.3333	33.70954	11.57078		Intercept		
0.15625	1369	1421	1410		125.6667	177.6667	166.6667	156.6667	27.40438	17.49216		-210.0106		
0.078125	1377	1448	1398		133.6667	204.6667	154.6667	164.3333	36.47373	22.19497	σ	S (slope)	LOD	LOQ
0.0390625	1245	1231	1259		1.666667	-12.33333	15.66667	1.666667	14	840	25.42309	988.5514	0.077153	0.257175
Bckgd	1221	1271	1238	1243.333	-22.33333	27.66667	-5.333333							

µM NaNO2	Corr.Fluor.	curve
20	20117	20.56
10	8878	9.19
5	4140	4.40
2.5	2120	2.36
1.25	1008	1.23
0.625	527	0.75
0.3125	291	0.51
0.15625	157	0.37
0.078125	164	0.38
0.0390625	2	0.21

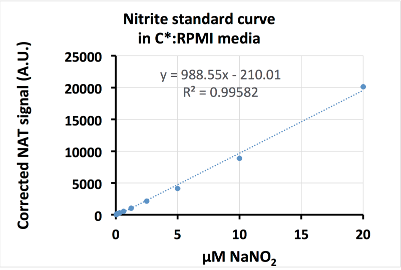

MSC media

µM NaNO2	i	ii	iii		i	ii	iii	ave	sd	cv				
20	48606	46345	47238		47048.67	44787.67	45680.67	45839	1138.785	2.484316				
10	25414	25831	26340		23856.67	24273.67	24782.67	24304.33	463.7611	1.908142				
5	13963	14469	13940		12405.67	12911.67	12382.67	12566.67	299	2.37931				
2.5	7721	7601	7442		6163.667	6043.667	5884.667	6030.667	139.9536	2.320698				
1.25	4183	4401	4410		2625.667	2843.667	2852.667	2774	128.5392	4.633714				
0.625	2804	2687	2933		1246.667	1129.667	1375.667	1250.667	123.0488	9.838654				
0.3125	1902	1915	2027		344.6667	357.6667	469.6667	390.6667	68.72409	17.59149		Intercept		
0.15625	1566	1620	1620		8.666667	62.66667	62.66667	44.66667	31.17691	69.79906		-17.59699		
0.078125	1366	1387	1463		-191.3333	-170.3333	-94.33333	-152	51.03267	-33.57412	σ	S (slope)	LOD	LOQ
0.0390625	1430	1427	1498		-127.3333	-130.3333	-59.33333	-105.6667	40.15387	-38.00051	63.3114	2330.25	0.081508	0.271694
Bckgd	1489	1614	1569	1557.333	-68.33333	56.66667	11.66667							

µM NaNO2	Corr.Fluor.	curve
20	45839	19.68
10	24304	10.44
5	12567	5.40
2.5	6031	2.60
1.25	2774	1.20
0.625	1251	0.54
0.3125	391	0.18
0.15625	45	0.03
0.078125	-152	-0.06
0.0390625	-106	-0.04

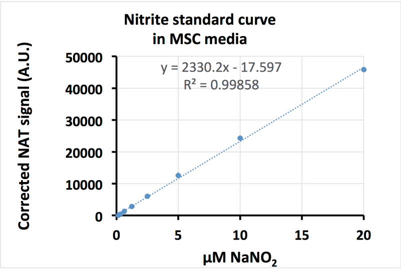

Supplement: S1 Protocol — (PDF) [file pone.0163735.s007.pdf]
